# Ultrastrong TEMPO‐Oxidized Densified Bamboo via Interface Decoupling and Hierarchical Toughening

**DOI:** 10.1002/advs.202521841

**Published:** 2026-03-12

**Authors:** Ziyu Ba, Hongyun Luo, Jie Cui, Juan Guan, Zhaoliang Guo, Robert O. Ritchie

**Affiliations:** ^1^ School of Materials Science and Engineering Beihang University Beijing P. R. China; ^2^ School of Materials Science and Engineering, Beijing Advanced Innovation Centre for Biomedical Engineering Beihang University Beijing P. R. China; ^3^ Department of Materials Science & Engineering University of California Berkeley California USA

**Keywords:** densified bamboo, hydrogen bonds, multi‐scales, strengthening mechanisms, TEMPO oxidized

## Abstract

The mechanical potential of natural fiber‐based composites remains restricted by interfacial constraints, preventing them from robust structural applications. Here, an interface decoupling strategy based on selective TEMPO (2,2,6,6‐tetramethylpiperidine‐1‐oxyl) oxidation is applied to enhance the mechanical properties of densified bamboo simultaneously with thermally‐assisted structural densification. The resulting compact TEMPO‐oxidized densified bamboo exhibits ultrahigh tensile strength of 661 MPa and toughness of 22 MJ m^−3^, representing 5.5 times higher than natural bamboo. Fracture morphologies, characterized by extensive fibril pull‐out and bridging that enhance energy dissipation, align with acoustic emission data revealing frequent, low‐energy microdamage events and a continuous, non‐catastrophic failure process. Specifically, TEMPO‐induced carboxylation disrupts orderly hydrogen bonds and increases the relative contribution of van der Waals interactions at the molecular level, enabling stress dissipation through a network of weaker, more reversible intermolecular forces. This work demonstrates that hierarchical interface engineering offers a broadly applicable strategy to endow natural composites with toughness and strength far exceeding their original mechanical paradigm.

## Graphical abstract

This study develops TEMPO‐oxidized densified bamboo that achieves ultrahigh strength and toughness by decoupling rigid hydrogen bonds into reversible van der Waals interactions. TEMPO oxidation reorganizes the cellulose hydrogen‐bonding network, facilitating energy dissipation via reversible interactions, thereby establishing a general strategy to advance natural composites beyond conventional mechanical limits.

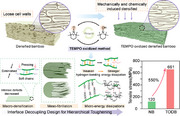

## 1 Introduction

Natural materials are receiving increasing attention as sustainable structural candidates owing to their biodegradability, broad availability, and low processing demands [1]. Among them, bamboo stands out due to its rapid growth, global abundance, and high mechanical strength [2, 3] (typically 100–200 MPa in tension and bending), which has enabled its use in both traditional and modern structural applications. These advantageous properties arise from a hierarchical architecture dominated by cellulose [4], whose crystalline nanofibrils (the glucan chains within fibrils adopt an ordered arrangement) exhibit exceptional intrinsic properties, including axial tensile strength up to 7 GPa and a modulus exceeding 100 GPa [5, 6]. However, such remarkable nanoscale performance is not fully translated into the macroscale behavior of native bamboo, indicating that its mechanical potential remains significantly underutilized. This disparity is primarily attributed to two factors: the presence of non‐load‐bearing components such as lignin and hemicellulose [7, 8], which reduce stiffness and hinder stress transfer between cellulose microfibrils, and an inherently porous cellular structure that undermines structural integrity and promotes stress concentrations [9].

Common approaches include resin infiltration [10] and modification with polymers [11] or nanoparticles [12], which primarily enhance environmental durability under moisture or chemical exposure, but yield only modest improvements in mechanical strength. In contrast, top‐down densification has become the prevailing strategy for enhancing mechanical performance [13]. This approach typically involves alkali pretreatment to remove lignin and hemicellulose, followed by microwave drying [14] or hot pressing [15], which improves material compactness and increases the relative content of load‐bearing cellulose. As a result, the mechanical strength of densified bamboo can be as high as 770 MPa, more than twice of their native bamboo (∼300 MPa), reaching values comparable to structural metals such as steel and aluminum. However, most commonly used bamboo exhibits tensile strengths below 150 MPa [16, 17]. For such bamboo, conventional hot‐press densification generally increases the strength only to around 300 MPa [18, 19, 20, 21], which remains far lower than that of metallic structural materials. Therefore, further enhancement of mechanical performance is required to enable the broader structural application of bamboo.

**FIGURE 1:**
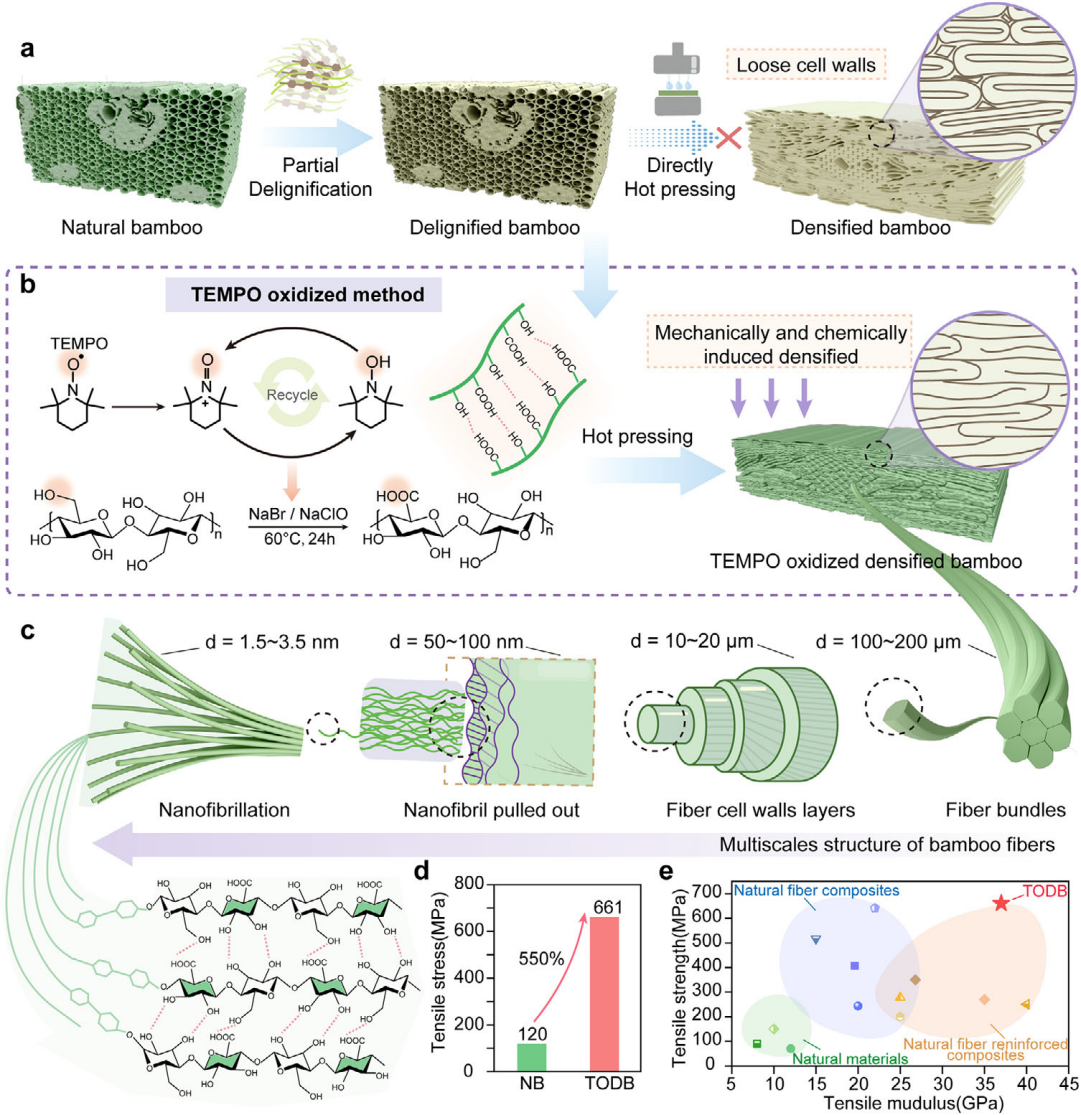
Schematic diagram of processing approaches of TEMPO‐oxidized densified bamboo material and its multiscale structure. (a) Preparation of delignified and densified bamboo materials. (b) Recyclable oxidation system used for preparing TEMPO‐oxidized densified bamboo. (c) Multiscale structure with corresponding dimensional ranges of bamboo fibers. (d) Tensile strength comparison between natural bamboo and TEMPO‐oxidized densified bamboo. (e) Tensile strength versus tensile stiffness of TEMPO‐oxidized densified bamboo compared to other materials (natural materials, natural fiber composites, and natural fiber reinforced composites). Detailed data for all plotted materials were provided in the Supporting Information.

Moreover, residual microscale porosity and local heterogeneity remain (Figure 1a), limiting the full utilization of cellulose's intrinsic strength. These structural imperfections largely originate from inadequate control over interfacial and molecular interactions during the densification process, and call for interfacial strategies that precisely regulate molecular interactions to minimize structural defects.

Recent studies have shown that chemical modulation of cellulose interfaces offers more precise structural control than physical densification alone. One particularly effective method is TEMPO (2,2,6,6‐tetramethylpiperidine‐1‐oxyl) oxidation [22, 23], a mild, aqueous, and environmentally benign process that selectively introduces carboxyl groups at the C6 position of glucose units, especially in amorphous regions, without disrupting the crystalline cellulose framework [24, 25]. Its ability to enhance surface reactivity while preserving the polymer backbone makes it a highly versatile tool for tailoring cellulose‐based materials.

Interestingly, beyond its conventional role in nanofiber production, TEMPO‐treated cellulose has been observed to undergo spontaneous shrinkage during ambient drying, resulting in densified structures with markedly reduced porosity and significantly enhanced mechanical properties in thin films [26, 27]. However, this densification behavior has been primarily demonstrated in membrane‐like materials, and its applicability to bulk systems remains uncertain. The physical and chemical mechanisms underlying this self‐shrinking phenomenon are not yet fully understood, particularly with regard to the contributions of capillary forces and chemically‐induced molecular interactions [28].

In parallel, oxidized cellulose composites have exhibited distinct fracture behavior, including increased fiber pull‐out and interfacial bridging, indicative of modified damage evolution at the microscale [29]. These observations imply that TEMPO oxidation holds potential for tailoring mechanical behavior across multiple length‐scales in cellulose‐based systems [30, 31]. To explore this potential, we conducted a preliminary study applying TEMPO oxidation to bulk bamboo scaffolds, followed by hot pressing, achieving a tensile strength of 661 MPa. This result demonstrates the capability of combining chemical modification with densification to enhance the mechanical performance of bulk bamboo. Indeed, addressing the associated structural evolution and fracture mechanisms is essential for advancing interfacial engineering in hierarchical natural materials and developing high‐performance bio‐derived composites.

Herein, a recyclable TEMPO‐mediated oxidation system was employed to further modify the delignified bamboo scaffold, taking advantage of the alkaline environment established during alkali treatment. The oxidized scaffold was then densified through hot pressing to fabricate TEMPO‐oxidized densified bamboo (TODB) (Figure 1b). This process effectively eliminated the intrinsic structural defects present in conventional densified bamboo (DB) and generated a well‐organized hierarchical fiber structure (Figure 1c), which significantly contributed to a multiscale toughening effect in TEMPO‐oxidized densified bamboo. Mechanical testing revealed a remarkable enhancement in tensile performance, with the tensile strength of TEMPO‐oxidized densified bamboo increasing by approximately 550% compared to that of natural bamboo (NB) (Figure 1d). In addition, both the tensile strength and modulus of TEMPO‐oxidized densified bamboo exceeded those of typical natural fiber‐reinforced composites reported in the literature (Figure 1e and Table S1). To elucidate the enhancement, strengthening and toughening mechanisms were investigated across multiple length‐scales, from molecular‐level interactions and nanoscale structure to macroscopic fracture behavior. This work emphasizes the critical role of interfacial design in enabling effective energy dissipation across scales, offering a promising strategy for developing ultrastrong and toughened bamboo‐based structural materials.

## 2 Results and Discussion

### 2.1 Chemical Characterization and Molecular‐Level Structural Reconstruction of TEMPO‐Oxidized Densified Bamboo

**FIGURE 2:**
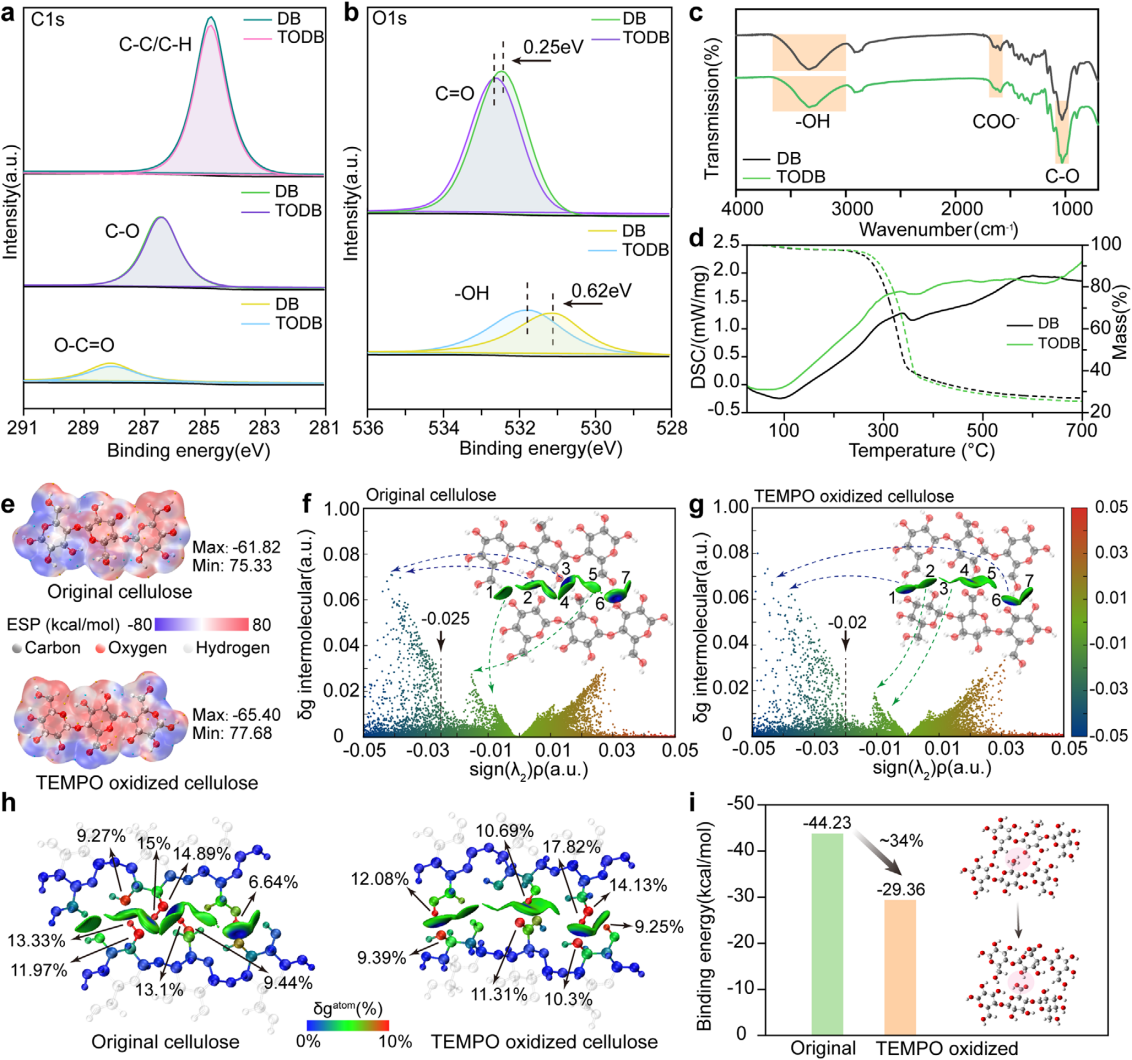
Chemical characterization of TEMPO‐oxidized densified bamboo. X‐ray photoelectron spectroscopy high‐resolution spectra of (a) C 1s and (b) O 1s peaks of densified bamboo and TEMPO‐oxidized densified bamboo. (c) FTIR spectra of densified bamboo and TEMPO‐oxidized densified bamboo. (d) DSC/TG analysis of densified bamboo and TEMPO‐oxidized densified bamboo. (e) Electrostatic potential (ESP) maps of original cellulose (top) and TEMPO‐oxidized cellulose (bottom). Independent gradient model based on Hirshfeld partition (IGMH) scatter plots and isosurface visualizations for (f) original cellulose and (g) TEMPO‐oxidized cellulose. Scatter plots show sign(λ_2_)ρ versus δg‐inter, where negative values indicate attractive interactions (e.g., hydrogen bonding), and positive values indicate steric repulsion. (h) Atomic contributions to noncovalent interactions (δg‐atom) based on IGMH analysis, with color from green to red indicating higher contributions. (i) Binding energy comparison of original and TEMPO‐oxidized cellulose.

TEMPO oxidation enables the selective conversion of primary hydroxyl groups on cellulose C6 sites into carboxyl functionalities, offering a controlled route to tailor interfacial chemistry without disrupting the crystalline framework. Compositional analysis [32] (Figure S1) shows a slight increase in cellulose content after TEMPO oxidation. To further investigate the chemical changes induced by TEMPO oxidation, high‐resolution X‐ray photoelectron spectroscopy (XPS) was performed on both densified bamboo and TEMPO‐oxidized densified bamboo samples (Figure 2a,b). The C 1s spectra show only minor changes in peak intensity after TEMPO treatment, suggesting that the cellulose carbon backbone is largely preserved. In contrast, the O 1s spectrum of the TODB sample showed a clear shift toward higher binding energy, with the C─O and −OH peaks shifting by 0.25 and 0.62 eV. This shift is primarily attributed to the introduction of electron‐withdrawing carboxyl groups, which modify the local electron density around oxygen atoms and result in an increased binding energy [33]. Such a redistribution of electron density, together with the moderate reduction in lignin content after TEMPO oxidation, is expected to influence local interfacial interactions, potentially weakening lignin‐mediated bonding and inducing interlamellar decoupling.

Employing Fourier transform infrared (FTIR) spectroscopy (Figure 2c) indicated that the TEMPO‐oxidized densified bamboo spectrum exhibited more pronounced absorption features at ∼1600 and ∼1720 cm^−1^, corresponding to the asymmetric stretching vibrations of carboxylate (−COO^−^) and the C═O stretching of carboxylic acid groups [34], respectively. Moreover, the −OH stretching band in the range of 3300−3400 cm^−1^ appears relatively weaker for TODB compared to DB, suggesting a modification of the hydrogen‐bonding environment [35]. Thermal studies by differential scanning calorimetry and thermogravimetric (DSC/TG) analysis (Figure 2d) revealed that TEMPO‐oxidized densified bamboo exhibited a slightly higher onset decomposition temperature compared to conventional densified bamboo, indicating improved thermal stability after TEMPO oxidation. The reduced low‐temperature weight loss also suggests a decrease in loosely bound water, likely resulting from the denser structure and weaker hydrogen bonding network in TEMPO‐oxidized densified bamboo. Overall, the oxidation of TEMPO introduced oxygen‐containing functional groups while maintaining the structural integrity of the cellulose, thus providing a chemical basis for TEMPO‐oxidized densified bamboo.

To further elucidate the effects of TEMPO modification on intermolecular interactions, theoretical simulations were conducted. As shown in Figure 2e and Figure S2, electrostatic potential (ESP) mapping [36] revealed that TEMPO oxidation led to more pronounced positive and negative extrema on the cellulose surface, with ESP values shifting from +75.33 to +77.68 kcal/mol and −61.82 to −65.40 kcal/mol, respectively. The increased local charge separation indicates enhanced surface polarity due to the introduction of electron‐withdrawing carboxyl groups, which may facilitate stronger intermolecular interactions such as hydrogen bonding and dipole–dipole attraction. Additionally, the independent gradient model, based on Hirshfeld partition scatter plots [37] in Figure 2f,g, visualizes the distribution of intermolecular interactions between cellulose chains before and after TEMPO oxidation. The sign(λ_2_)ρ analysis enables differentiation between attractive and repulsive interactions, where negative values correspond to hydrogen bonding and positive values to steric effects. Notably, after oxidation, the blue‐shaded data points representing hydrogen bonds became more diffuse (Figure 2g), which means a more heterogeneous distribution of hydrogen bonding interactions. This trend aligns with the observed slight decrease in electron density at the bond critical points (ρ(BCP)), from 0.025 to 0.020, indicating a redistribution of intermolecular interaction strength. The isosurface visualizations further illustrate that TEMPO oxidation leads to a rearrangement of the weak interaction network between cellulose molecules. Specifically, the strength of hydrogen bonds has been calculated [38], where it was found that hydrogen bond regions 3 and 5 in the original structure were weakened or replaced by van der Waals interactions in the oxidized form (Figures S3 and S4 and Table S2); this clearly reveals that TEMPO‐induced carboxylation alters the spatial distribution of hydrogen bonds, disrupting previous hydrogen‐bonding regions and creating new polar interaction domains. While atoms involved in weak interactions in original cellulose contribute in a relatively uniform and dispersed manner, consistent with a stable hydrogen‐bonding network, the contributions after TEMPO oxidation become more concentrated around specific regions such as carboxyl groups, with a peak value reaching 17.82% (Figure 2h). The binding energy decreased from −44.23 to −29.36 kcal/mol after TEMPO oxidation, as intermolecular interactions weakened following hydrogen bond disruption and network reorganization.

Taken together, the results demonstrate that TEMPO oxidation selectively modifies the molecular interface of cellulose, introducing functional groups without disrupting the backbone structure. The resulting rearrangement of the hydrogen‐bonding network weakens overall intermolecular interactions.

### 2.2 Structural Reorganization and Densification Behavior Driven by TEMPO Oxidation

**FIGURE 3:**
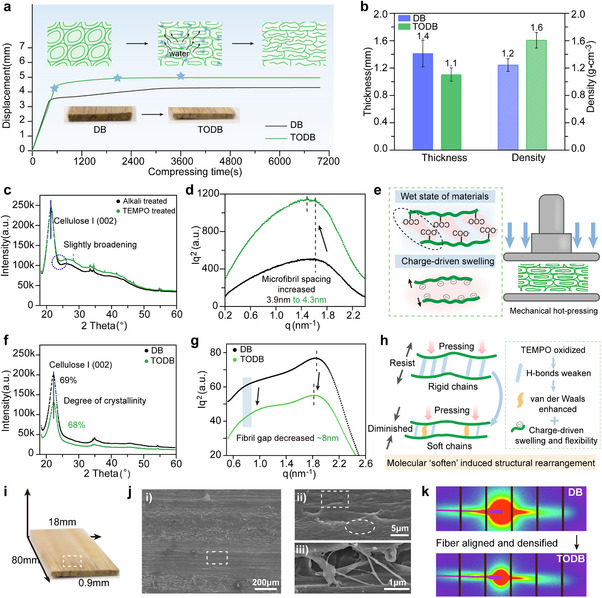
Structural changes and densification behavior of TEMPO‐oxidized densified bamboo. (a) Compression displacement over time during hot pressing of densified bamboo and TEMPO‐oxidized densified bamboo. (b) Comparison of thickness and density between densified bamboo and TEMPO‐oxidized densified bamboo. (c) WAXS patterns and (d) SAXS Kratky plots of wet state alkali‐treated and TEMPO‐oxidized bamboo samples before densification. (e) Schematic illustration of increased microfibril spacing induced by TEMPO oxidation through charge‐driven swelling. (f) WAXS patterns and (g) SAXS Kratky plots of densified bamboo and TEMPO‐oxidized densified bamboo (TODB). (h) Schematic of structural rearrangement mechanism during densification. (i) Schematic of the TODB specimen. (j) Scanning electron microscopy (SEM) images of the TODB at different magnifications: i) low magnification showing overall morphology, (ii, iii) high magnification revealing interfacial features and nanofibrillar bridging. (k) 2D SAXS patterns of densified bamboo and TODB.

TEMPO oxidation markedly alters the densification behavior of bamboo. This is explored in Figure 3, where the influence of such oxidation is examined on the structural evolution and densification behavior of conventional densified bamboo and TODB during hot pressing. Under identical compressive stress during hot pressing, TEMPO‐oxidized bamboo displayed a larger displacement over time as moisture was gradually removed (Figure 3a). As a result, the final thickness of the TEMPO‐oxidized densified bamboo sample reduced from 1.4 to 1.1 mm (Figure 3b), consistent with previous reports on the self‐densification behavior of TEMPO‐oxidized cellulose systems [26, 27]. To investigate the changes in bamboo cellulose fibers, specimens in the wet state were tested prior to hot pressing. Both alkali‐treated and TEMPO‐oxidized bamboo exhibited the (002) peak of cellulose I in the wide‐angle X‐ray scattering (WAXS) patterns (Figure 3c). However, the TEMPO‐treated sample showed slight broadening on the right shoulder of the (002) peak, indicating localized structural disorder at the crystalline‐amorphous interface, likely resulting from carboxylation‐induced swelling and hydrogen bond rearrangement [39]. Small‐angle X‐ray scattering (SAXS) Kratky plots (Figure 3d) revealed a notably higher peak intensity in the TEMPO‐treated bamboo compared to the alkali‐treated sample. Such an increase indicates a greater abundance of nanoscale voids within the microfibril bundles, particularly sized around ∼4 nm, likely arising from electrostatic repulsion between negatively‐charged carboxyl groups introduced during TEMPO oxidation [25], which drives charge‐induced swelling of cellulose microfibrils [40] (Figure 3e).

After densification, the cellulose structure was well preserved in both densified bamboo and TEMPO‐oxidized densified bamboo (Figure 3f), with the lower intensity of the SAXS Kratky plots for TODB compared to densified bamboo (Figure 3g) revealing a significant reduction in nanoscale voids. Whereas densified bamboo showed a broad shoulder at ∼0.8 nm^−1^, corresponding to the presence of ∼8 nm inter‐fibrillar gaps, this feature was absent in TEMPO‐oxidized densified bamboo. Instead, TODB exhibited a sharper peak at ∼3.5 nm, indicating tighter microfibril packing and substantially reduced porosity (Figure 3h). These results suggest that TEMPO oxidation promotes a more homogeneous and flexible rearrangement of the cellulose network during compaction, consistent with the charge‐driven swelling and molecular softening effects illustrated in Figure 3h. The formation of large fibrillar voids in conventional densified bamboo is likely attributed to limited molecular mobility during densification. The rigid hydrogen‐bonded network in untreated cellulose constrains structural rearrangement, leading to localized separation under compressive stress. In contrast, TEMPO oxidation partially disrupts hydrogen bonding and softens the cellulose matrix, enabling more homogeneous compaction and effective suppression of void formation. These results demonstrate that TEMPO oxidation not only facilitates macroscopic densification but also markedly improves nanoscale compactness.

As illustrated schematically in Figure 3h, the improved compaction in TEMPO‐oxidized densified bamboo could be attributed to the combined effects of weakened interfibrillar interactions and enhanced chain flexibility. Specifically, the partial disruption of hydrogen bonds and the introduction of surface carboxyl groups lead to increased electrostatic repulsion and local rearrangement of the intermolecular network. In regions where hydrogen bonding is diminished, van der Waals interactions become more dominant, providing weaker but longer‐range attractions that could allow cellulose fibrils to pack more closely during hot‐pressing‐induced compaction as moisture is expelled [41, 42]. Meanwhile, the disruption of amorphous domains by TEMPO oxidation increases the conformational mobility of cellulose chains, facilitating more efficient structural collapse under pressure. The enhanced softness at the molecular‐scale plays a decisive role in facilitating hierarchical compaction, bridging the chemical modification and structural densification from the chain level to the nanoscale.

### 2.3 Micro‐Mechanical Properties of TEMPO‐Oxidized Densified Bamboo

**FIGURE 4:**
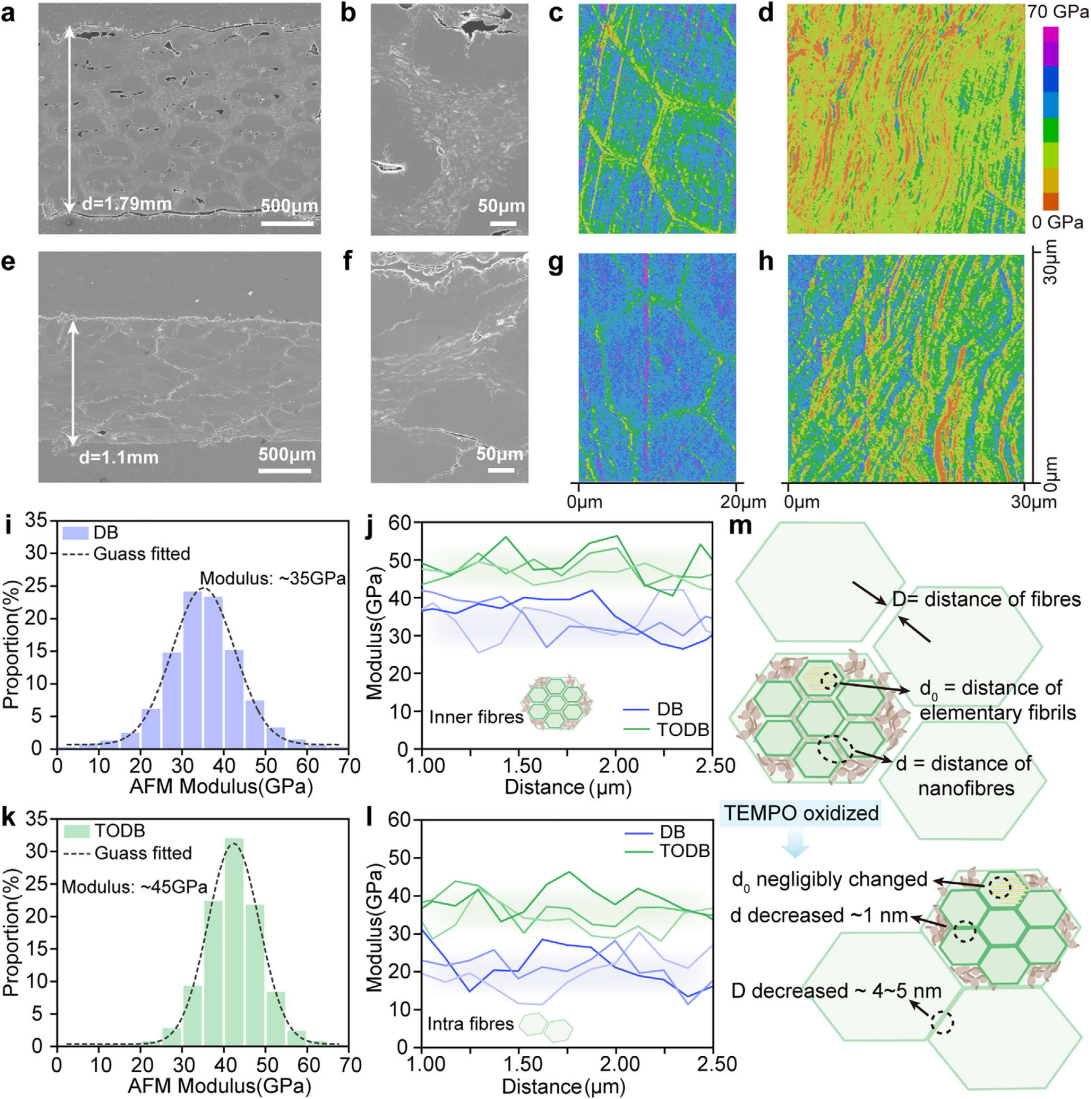
Morphological and micromechanical characterization of densified bamboo and TEMPO‐oxidized densified bamboo. Cross‐sectional SEM images of densified bamboo showing (a) the overall thickness and (b) higher‐magnification views of internal structures. AFM modulus maps of densified bamboo for (c) microfibril regions and (d) densified cell regions. Cross‐sectional SEM images of TODB showing (e) the overall thickness and (f) higher‐magnification views of internal structures. AFM modulus maps of TODB for (g) microfibril regions and (h) densified cell regions. (i) Histograms of AFM modulus distribution for densified bamboo fitted with Gaussian curves. (j) Line profiles of modulus along inner fiber regions. (k) Histograms of AFM modulus distribution for TODB fitted with Gaussian curves. (l) Line profiles of modulus along intra‐fiber regions. (m) Schematic illustration of hierarchical fiber spacing at three levels: nanofiber distance *d*, elementary fiber distance *d*_0_, and macroscopic fiber bundle spacing *D*.

Figure 3i–k reveals a compact morphology in TEMPO‐oxidized densified bamboo, characterized by well‐aligned fiber bundles, reduced interfacial voids, and the presence of nanofibrillar bridging. These multiscale structural features indicate stronger interfacial cohesion and more uniform fiber packing, both of which might contribute to improve mechanical performance of TEMPO‐oxidized densified bamboo. Figure 4a,b shows that although densified bamboo undergoes hot pressing, the rigid vascular bundles encapsulating large conduits are not fully collapsed (Figure 3h). The resulting macropores, typically several hundred micrometers in size, remain distributed throughout the structure, potentially diminishing compaction efficiency and promoting crack initiation under mechanical stress. In contrast, TODB shows no discernible macroscopic voids, reflecting the effective compaction of cell walls and vascular structures enabled by TEMPO‐induced softening of the cellulose matrix during hot pressing (Figure 4e,f).

To further examine the influence of this hierarchically densified structure on the micromechanical behavior of bamboo fibers, atomic force microscopy (AFM) was employed to assess the elastic modulus of densified bamboo and TEMPO‐oxidized densified bamboo. Modulus maps of densified bamboo and TEMPO‐oxidized densified bamboo (Figure 4c,d,g,h) reveal distinct stiffness contrasts between microfibers (∼20 µm) and surrounding parenchyma regions, with green and blue indicating low and high modulus zones, respectively. The corresponding histograms (Figure 4i,k) quantify the modulus distribution, enabling direct comparison between the two materials. Specifically, TODB shows consistently higher stiffness in both microfibers and parenchyma regions compared to densified bamboo, with the average microfiber modulus increasing from ∼35 to ∼45 GPa. Additionally, line profiling across ∼2 µm‐wide inter‐ and intra‐fiber zones further reveals that TEMPO‐oxidized densified bamboo consistently exhibits values over 10 GPa higher than densified bamboo, further confirming enhanced micromechanical performance at the microfiber scale (Figure S5).

Figure 4m illustrates the hierarchical fiber spacing of bamboo after TEMPO oxidation, including nanofiber spacing (*d*_0_), elementary fibril distance (*d*), and microfibril bundle spacing (*D*). The nanofiber spacing remains negligibly changed, as evidenced by the stable SAXS Kratky features (Figure 3d,g), indicating that the nanofiber framework is structurally preserved. Meanwhile, the elementary fibril spacing *d* decreases slightly by less than 1 nm, suggesting denser molecular packing. At the larger scale, *D* is reduced by approximately 4 nm, reflecting enhanced inter‐bundle compaction. This hierarchical densification effect underlies the observed enhancement in micromechanical performance, revealing the reinforcing role of interfacial softening and structural compaction across molecular and microscale levels.

### 2.4 Macro Mechanical Properties and Acoustic Emission Analysis of TEMPO‐Oxidized Densified Bamboo

**FIGURE 5:**
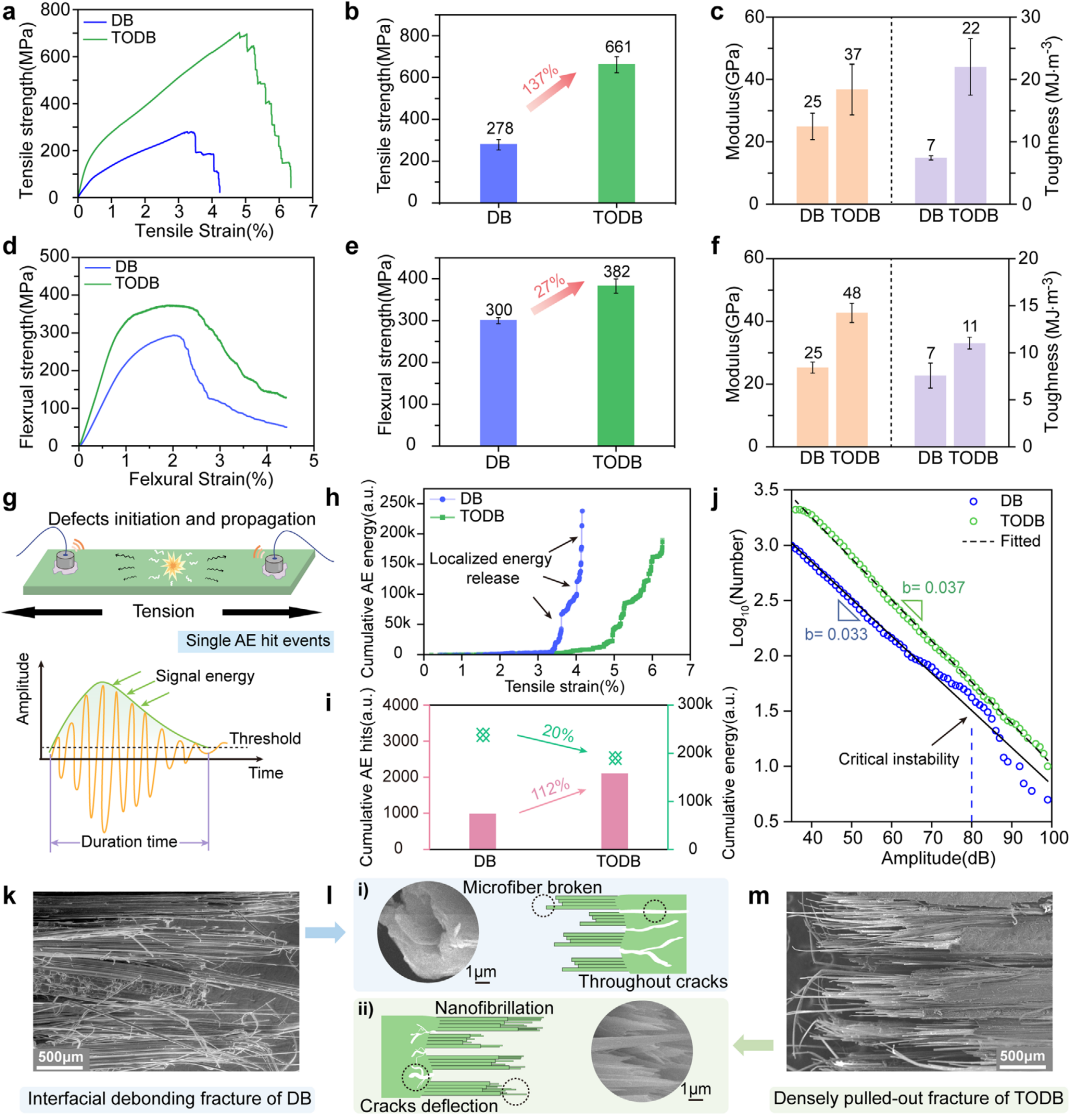
Macro‐mechanical performance and acoustic emission analysis of densified bamboo and TEMPO‐oxidized densified bamboo. (a) Tensile stress−strain curves of densified bamboo and TODB. (b) Comparison of tensile strength between densified bamboo and TODB. (c) Tensile modulus and toughness of densified bamboo and TODB. (d) Flexural stress−strain curves of densified bamboo and TODB. (e) Comparison of flexural strength between densified bamboo and TODB. (f) Flexural modulus and toughness of densified bamboo and TODB. (g) Schematic of the AE testing setup and definition of AE signal parameters, including amplitude threshold and duration of a single hit event. (h) Cumulative AE energy curves of densified bamboo and TODB during tensile loading. (i) Comparison of total cumulative AE hits and total AE energy of densified bamboo and TODB. (j) AE amplitude distribution fitted with a Gutenberg−Richter (GR) type power‐law model. (k) SEM of densified bamboo interfacial debonding fracture with limited fiber bridging and (m) TODB fracture of densely pulled‐out nanofibers, (l) shows schematic and high‐magnification images illustrating fracture mechanisms of (i) densified bamboo and (ii) TEMPO‐oxidized densified bamboo.

Figure 5 compares the tensile and flexural mechanical properties of densified bamboo and TEMPO‐oxidized densified bamboo. A remarkable enhancement in tensile performance is observed in TEMPO‐oxidized densified bamboo, with tensile strength rising from 278 to 661 MPa, corresponding to a 137% improvement (Figure 5b). The tensile modulus increases to 37 GPa, and toughness rises to 22 MJ m^−3^, representing more than a threefold enhancement (Figure 5c). This marked increase in tensile properties is attributed to the synergistic effects of TEMPO‐induced carboxylation and enhanced structural densification, which together reinforce the integrity of the bamboo composite. Notably, both tensile strength and modulus of TODB exceed those of the majority of natural fiber‐reinforced composites previously reported [13, 15, 19, 43] (Figure 1e), underscoring its potential as a high‐performance bio‐based structural material. In terms of flexural behavior, TEMPO‐oxidized densified bamboo also demonstrates substantial improvements. Its flexural strength reaches 382 MPa, reflecting a 27% increase over DB (Figure 5e), while the flexural modulus nearly doubles from 25 to 48 GPa (Figure 5f). These results further confirm the efficacy of TEMPO oxidation in improving both stiffness and toughness.

To further investigate the fracture behavior of TEMPO‐oxidized densified bamboo, acoustic emission (AE) analysis was performed during tensile testing (Figure 5g). As a non‐destructive and real‐time monitoring technique, AE captures transient elastic waves generated by microstructural events such as crack initiation, interfacial debonding, and fiber pull‐out, offering valuable insights into damage evolution and failure mechanisms in composite materials [44, 45, 46]. The cumulative AE energy recorded during tensile loading quantitatively reflects the progressive evolution of microstructural damage and serves as a sensitive indicator of fracture behavior. As shown in Figure 5h, densified bamboo exhibits a sudden rise in AE energy near failure, corresponding to localized damage accumulation and abrupt structural breakdown. In contrast, TODB accumulates AE energy more gradually throughout the loading process, indicative of distributed damage progression and improved fracture resistance. This distinction is further supported by the AE energy distributions shown in Figure S6. For densified bamboo, AE activity remains low until the final stage, where abrupt bursts of high‐energy signals emerge, highlighting a catastrophic failure mode. On the other hand, TEMPO‐oxidized densified bamboo displays a broader and more continuous spread of AE events over the strain range, with varied energy levels, reflecting a more progressive and energy‐dissipative fracture process. These observations reinforce the notion that TEMPO‐induced structural reorganization promotes distributed damage evolution and enhances the toughness of densified bamboo.

Quantitative analysis of AE activity (Figure 5i) shows that TEMPO‐oxidized densified bamboo produces more than twice the number of AE hits compared to densified bamboo, despite exhibiting an approximately 20% reduction in total AE energy. The increased hit count indicates a higher frequency of microstructural damage events during loading [44], whereas the reduced cumulative energy suggests that these events are more dispersed and individually less intense. This shift toward frequent but lower‐energy acoustic events reflects a more distributed and gradual failure mechanism in TODB, aligning with its superior fracture resistance. The AE amplitude distribution was analyzed using the Gutenberg−Richter (GR) type power‐law model [47], which is commonly used to describe the statistical behavior of fracture‐related events:

logN=a−bA

where *N* is the cumulative number of AE events with amplitude ≥*A*, and *b* is the slope reflecting the relative proportion of small versus large amplitude events. A higher *b*‐value indicates a greater prevalence of low‐amplitude, small‐scale events, characteristic of a more gradual and distributed failure process [48, 49]. As shown in Figure 5j, the *b*‐value for TEMPO‐oxidized densified bamboo (0.037) is slightly steeper than that of densified bamboo (0.033), suggesting a larger proportion of low‐energy damage events in the oxidized material. Furthermore, the AE amplitude distribution of TODB exhibits strong agreement with the fitted Gutenberg−Richter curve across the full amplitude range, while densified bamboo shows pronounced deviation above 70 dB, indicative of abrupt, high‐energy fracture events. This improved statistical conformity in TEMPO‐oxidized densified bamboo reflects a more stable damage evolution, underscoring the role of TEMPO oxidation in promoting distributed microfracture and enhancing fracture tolerance. The broader and more uniform distribution of average signal level (ASL) in TEMPO‐oxidized densified bamboo further supports this gradual and distributed fracture behavior (Figure S6). As shown in previous AE studies, which have identified characteristic peak‐frequency ranges for different failure modes [29], the TEMPO‐oxidized densified bamboo shows continuous medium‐frequency AE signals (200−250 kHz) throughout the tensile process, which correspond to progressive nanofiber debonding and pull‐out (Figure S7). In contrast, densified bamboo exhibits high‐frequency bursts (∼300 kHz) near final fracture, characteristic of sudden fiber bundle breakage. Together, these results indicate that TEMPO oxidation promotes stable, multiscale interfacial deformation dominated by nanofiber pull‐out, and that the associated change in fracture modes accounts for the strength enhancement beyond density effects via molecular‐level interfacial weakening and decoupling.

Fracture morphologies further elucidate the distinct failure mechanisms of densified bamboo and TEMPO‐oxidized densified bamboo. Figure 5k presents the tensile fracture surface of densified bamboo, characterized by a discontinuous and disintegrated morphology, reflecting uncontrolled crack propagation and compromised interfacial adhesion. TEMPO‐oxidized densified bamboo fracture surface (Figure 5m, Figures S8 and S9) remains largely intact, with numerous nanofibers (width below 100 nm) bridging the crack front, indicative of effective energy dissipation through fiber pull‐out and improved structural cohesion. Detailed SEM observations further reveal hierarchical deformation features, including nanoscale fibril bridging and microscale cell tearing and deflection, which provide additional pathways for crack deflection and energy absorption during fracture. High‐magnification views in Figure 5l further illustrate this distinction. Densified bamboo (Figure 5l(i)) exhibits limited deformation and fiber engagement, whereas TODB (Figure 5l(ii)) shows dense nanofibrillar pull‐out, providing microstructural evidence of a toughening mechanism enabled by hierarchical reinforcement and interfacial integrity. These morphological features align with the acoustic emission and mechanical data, reinforcing the role of TEMPO oxidation in promoting a more damage‐tolerant failure process.

### 2.5 Multiscale Synergistic Toughening Mechanism of TEMPO‐Oxidized Densified Bamboo

**FIGURE 6:**
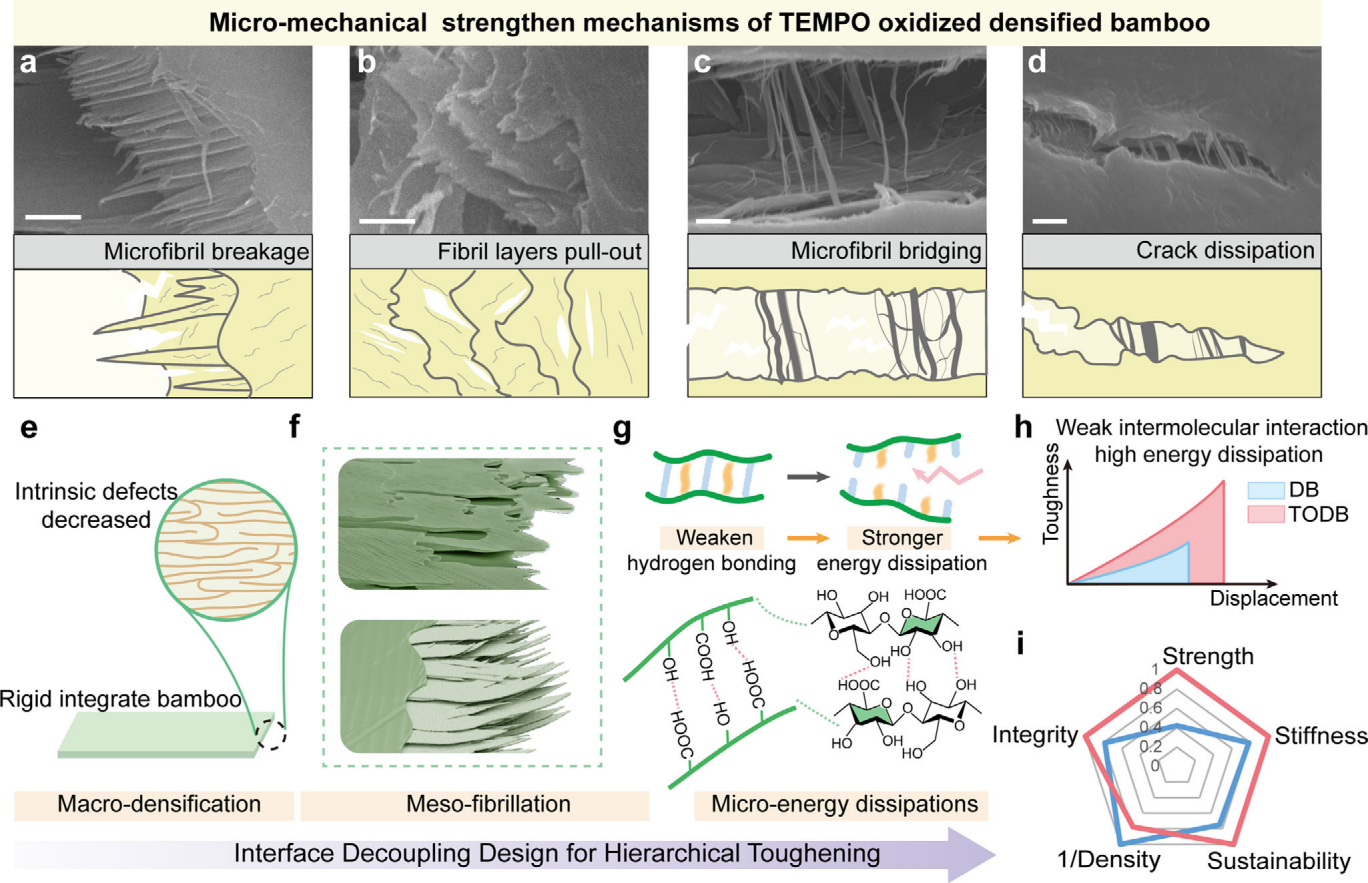
Multiscale synergistic toughening mechanism of TEMPO‐oxidized densified bamboo materials. Micromechanical toughening mechanisms of TEMPO‐oxidized densified bamboo, (a) microfibril breakage, (b) fibril layers pull‐out, (c) microfibril bridging, and (d) crack deflection; the scale bar is 1 µm. Multiscale synergistic toughening mechanism of TEMPO‐oxidized densified bamboo, including (e) macroscopic structural densification that enhances the integrity of bamboo, (f) meso‐scale fibrillation and fiber layer pull‐out that contributes to strengthening and toughening; and (g) molecular‐level energy dissipation. (h) Schematic illustration showing that weak intermolecular interactions in TEMPO‐oxidized densified bamboo lead to greater energy dissipation and improved toughness during deformation. (i) Radar chart comparing the overall performance (tensile strength and stiffness) of TEMPO‐oxidized densified bamboo and densified bamboo, all values are normalized to 0–1.

Toughening mechanisms of TEMPO‐oxidized densified bamboo were further elucidated by analyzing fracture surfaces at higher magnification. As depicted in Figure 6a–d, the crack front in TEMPO‐oxidized densified bamboo reveals several nanoscale toughening features, including microfibril fracture, fibril layer pull‐out, microfibril bridging, and crack deflection. These localized micromechanical processes represent extrinsic toughening mechanisms [9] that dissipate energy through interfacial debonding and frictional sliding, while simultaneously impeding crack propagation by increasing crack path tortuosity and promoting stress redistribution. The synergistic activation of these mechanisms plays a critical role in enhancing the fracture resistance and overall toughness of TEMPO‐oxidized densified bamboo.

To interpret the origins of this superior mechanical behavior of TEMPO‐oxidized densified bamboo, a multiscale synergistic toughening model is proposed (Figure 6e–g). At the macroscopic level, the enhanced densification achieved through TEMPO oxidation significantly reduces intrinsic defects and promotes a more cohesive bamboo architecture. The selective oxidation that introduces carboxyl groups increases the surface charge density and electrostatic repulsion of cellulose fibrils, which might induce charge‐driven swelling and partially weaken local hydrogen‐bond interactions. As a consequence, the fibrillar network could exhibit greater flexibility and rearrangement capability, enabling more efficient structural densification during hot pressing. At the mesoscale, toughening is mediated by hierarchical microfibril deformation and interfacial mechanisms, such as fiber layer pull‐out and microfibril bridging (Figure 6a,b), which enhance mechanical interlocking and promote crack deflection. On the molecular scale, the introduction of carboxyl groups disrupts native hydrogen bonding, increasing chain mobility and enabling localized slippage. This facilitates efficient energy dissipation during deformation while maintaining overall structural coherence. To further support this multiscale toughening mechanism, an Ashby‐type strength‐density analysis is shown in Figure S10. Using the natural bamboo to densified bamboo transition as a baseline, the strength predicted from density scaling alone remains lower than the experimental value of TEMPO‐oxidized densified bamboo. This indicates that the improved mechanical performance cannot be explained by densification alone, but is closely related to the interfacial weakening and fracture mode transition discussed above.

Taken together, the synergistic interaction across molecular, microscopic, and macroscopic length‐scales results in a gradual and energy‐dissipative failure mode (Figure 6h), in stark contrast to the abrupt damage observed in untreated samples. As shown in the radar chart (Figure 6i), TEMPO‐oxidized densified bamboo achieves outstanding performance across key mechanical metrics such as strength, stiffness, and toughness, while maintaining its intrinsic sustainability. These findings highlight the efficacy of TEMPO oxidation not only as a chemical modification strategy but also as a structural design principle for developing high‐performance, damage‐tolerant bamboo‐based composites.

## 3 Conclusions

In summary, this study demonstrates the exceptional mechanical performance of TEMPO‐oxidized densified bamboo, enabled by interfacial design and hierarchical toughening. Through selective TEMPO‐induced carboxylation, the local hydrogen‐bond network is partially weakened, making the longer‐range van der Waals interactions relatively more prominent. This molecular‐level modulation reduces interfibrillar stiffness, enhances chain mobility, and facilitates structural rearrangement, allowing efficient densification during thermal compression. As a result, TEMPO‐oxidized densified bamboo achieves a tensile strength of 661 MPa, representing 550% and 137% improvements compared to natural and conventional densified bamboo, respectively, and outperforms most reported natural fiber‐reinforced composites. Real‐time acoustic emission analysis reveals a shift from catastrophic failure in densified bamboo to progressive, distributed damage in TEMPO‐oxidized densified bamboo, characterized by a 112% increase in AE hit count and a 20% reduction in total AE energy, indicating more frequent but less intense damage events. Fracture analysis reveals that the exceptional toughness arises from a multiscale mechanism beginning at the molecular level, where TEMPO‐induced carboxylation lowers local binding energy and partially weakens the rigid hydrogen‐bond network, allowing van der Waals interactions to play a more prominent role. This molecular softening promotes chain mobility and localized energy dissipation. At the microscale, such interfacial modifications give rise to dense nanofibril pull‐out and bridging, which impede crack propagation and further absorb energy. Macroscopically, the enhanced compaction achieved by reduced intermolecular stiffness ensures structural continuity and suppresses defect‐driven failure. These findings form an integrated, hierarchical toughening strategy, demonstrating that targeted molecular interfacial weakening can synergistically enhance both strength and toughness while maintaining the sustainability and structural integrity of bio‐based composites.

## 4 Methods

### 4.1 Materials

Five‐year‐old Moso bamboos (Phyllostachys edulis) were collected from the Hunan Province, China. The culms were harvested at approximately 1.5 m above the ground and processed into cuboid specimens with dimensions of 80 mm × 18 mm × 6 mm for natural bamboo. The bamboo was air‐dried prior to use, and the density under stable dry conditions was 0.66 g cm^−3^. The 2,2,6,6‐tetramethylpiperidine‐1‐oxyl (TEMPO, 99%), Sodium hydroxide (NaOH, AR, 96%), Sodium bromide (NaBr, AR, 99%), Sodium hypochlorite (NaClO, AR, 10%), and Sodium sulfite (Na_2_SO_3_, AR, 98%) were purchased from Aladdin Reagent Co., Ltd. and used as received without further purification.

### 4.2 Preparation of TEMPO‐Oxidized Densified Bamboo

Delignified bamboo samples were prepared by boiling Moso bamboo in a solution of 2.5 M NaOH and 0.4 M Na_2_SO_3_ for 8 h, followed by thorough washing with deionized water to remove residual alkali until the pH reached approximately 10. The delignified bamboo samples were then oxidized in a NaBr/NaClO/TEMPO system. The bamboo samples were immersed in 500 mL deionized water containing 0.2 mmol/g of TEMPO and 1.0 mmol/g of NaBr. Then, NaClO of 2.0 mmol/g was added dropwise, while the pH was maintained at 10 by continuous addition of 0.5 M NaOH. The oxidation was carried out at 60°C for 24 h without stirring to preserve the bamboo structure. After the reaction, the samples were washed thoroughly with deionized water until the pH was neutral. Finally, the bamboo samples were subjected to hot‐pressing at a compressive pressure of 6.5 MPa for 24 h to acquire TEMPO‐oxidized densified bamboo. Delignified bamboo samples without TEMPO treatment underwent the same hot‐pressing procedure to acquire densified bamboo (DB).

### 4.3 Characterization

X‐ray photoelectron spectroscopy (XPS, Thermo Fisher Scientific, USA) was performed using a K−Alpha spectrometer with radiation to analyze the chemical composition changes. The spot size was 400 µm, and the energy step size was 0.1 eV. Fourier transform infrared (FTIR) spectra were recorded using a Nicolet 6700 spectrometer (Thermo Fisher Scientific, USA) in attenuated total reflectance (ATR) mode over the range of 4000−400 cm^−1^. Differential scanning calorimetry and thermogravimetric analysis (DSC and TG) were analyzed using a STA449F3 instrument (NETZSCH, Germany) from ambient temperature to 700°C at a heating rate of 10°C min^−1^ under an air atmosphere. The density of the bamboo samples was calculated by measuring the mass and geometric volume of the specimens; at least six specimens were tested for each group to ensure statistical reliability. Simultaneous small‐angle and wide‐angle X‐ray scattering (SAXS/WAXS) measurements were carried out at the BL19U2 BioSAXS beamline of the Shanghai Synchrotron Radiation Facility (SSRF) to investigate the evolution of microstructural features in bamboo. The incident X‐ray wavelength was 1.033 Å. SAXS data were collected using a Pilatus 3 M detector positioned at a sample‐to‐detector distance of 2770 mm, while WAXS data were recorded with a Pilatus 300KW detector. The 2D scattering patterns were processed using Fit2D (v18.002) and RAW (v2.2.2). The microstructure and fracture morphology of bamboo samples were observed using a scanning electron microscope (SEM, JSM‐6010, JEOL Ltd., Japan) at an accelerating voltage of 15 kV. Atomic force microscopy (AFM, Dimension FastScan, Bruker Scientific Instruments, Germany) was performed to characterize the nanoscale mechanical properties and surface morphology of the modified bamboo samples. PeakForce quantitative nanomechanical mapping (PF‐QNM) mode was used to measure the DMT modulus.

### 4.4 Mechanical Properties Measurement

Tensile and flexural tests were conducted on a universal testing machine (Instron 5966, Instron Corporation, USA) equipped with a 10 kN load cell. Uniaxial tensile testing was carried out at a displacement rate of 1 mm min^−1^. Unnotched specimens with dimensions of 80 mm × 5 mm (length×width) were flexurally tested, with both ends of the specimens clamped between aluminum alloy tabs to ensure fracture occurred in the gauge section. Three‐point bending tests were performed at a displacement rate of 2 mm min^−1^.

### 4.5 Acoustic Emission Setup

Acoustic emission (AE) monitoring was employed to capture damage‐related acoustic signals during mechanical tests. Two piezoelectric sensors (Nano‐30, resonance frequency ∼140 kHz) were used for signal collection. A thin layer of Vaseline was smeared to enhance signal transmission between the specimen surface and the sensors. The signals were processed using a digital signal processor and recorded with an AEwin v3.61 acquisition system (Physical Acoustic Corporation, USA). An amplitude threshold of 35 dB was set to eliminate environmental background noise.

### 4.6 Quantum Chemical Calculations

All quantum chemical calculations were performed using Gaussian 16 [50] to explore the microscopic interaction changes of cellulose molecules before and after TEMPO oxidation. Density functional theory (DFT) calculations employed the B3LYP functional with D3(BJ) dispersion correction to account for noncovalent interactions [51, 52]. Geometry optimizations and frequency analyses were conducted using the def2‐SV(P) basis set, while single‐point energy refinements used the def2‐TZVP basis set [53] with basis set superposition error (BSSE) correction. Solvent effects were considered using the solvation model based on density (SMD) implicit solvation model with water as the solvent [54]. Wavefunction analyses were performed using Multiwfn program [55, 56] to investigate electronic structures and noncovalent interactions. Hydrogen bond energies were estimated from electron density descriptors. Molecular structures and interaction isosurfaces were visualized with Visual Molecular Dynamics (VMD)1.9.3 [57].

### 4.7 Statistical Analysis

Data are shown as standard deviation (SD) ± mean. Error lines in various data plots indicate SD for at least three tests on each sample. All data were processed using Origin 2018.

## Author Contributions

Z.Y.B. and H.Y.L. conceived the concept and designed the experiments. Z.Y.B., J.C., and Z.L.G. carried out the mechanical tests and characterization tests. Z.Y.B. carried out the computational simulation, analyzed the results, and wrote the initial draft of the paper. H.Y.L., J.G., and R.O.R were involved in the supervision of the work, discussion of the results, and revision of the manuscript. All the authors edited and reviewed the final manuscript.

## Funding

This work was supported by the National Key Research and Development Program of China (No. 2024YFC3013700) and the program of Ministry of Industry and Information Technology of P. R. China (J2019‐VI‐0001‐0114) to H.Y.L.

## Conflicts of Interest

The authors declare no conflicts of interest.

## Supporting information




**Supporting File**: advs74785‐sup‐0001‐SuppMat.docx.

## Data Availability

The data that support the findings of this study are available from the corresponding author upon reasonable request.
