# Supplementary material for: Ultrastrong TEMPO‐Oxidized Densified Bamboo via Interface Decoupling and Hierarchical Toughening
Source: Adv Sci (Weinh). 2026 Mar 12;13(29):e21841. doi: 10.1002/advs.202521841 (PMC13205609; doi:10.1002/advs.202521841)
Supplement: Supplementary file 1 — Supporting File: advs74785‐sup‐0001‐SuppMat.docx. [file ADVS-13-e21841-s001.docx]

**Supplementary Information**

**Ultrastrong TEMPO-Oxidized Densified Bamboo via Interface Decoupling and Hierarchical Toughening**

Ziyu Ba^1^, Hongyun Luo ^1, 2, 3, *^, Jie Cui^1^, Juan Guan^1, 2^, Zhaoliang Guo^1^,

Robert O. Ritchie^4, *^

^1^ School of Materials Science and Engineering, Beihang University, Beijing, P. R. China.

^2^ Beijing Advanced Innovation Centre for Biomedical Engineering, Beihang University, Beijing, P. R. China.

^3^ Beijing Key Laboratory of Advanced Nuclear Materials and Physics, Beihang University, Beijing, P. R. China.

^4^ Department of Materials Science & Engineering, University of California, Berkeley, CA 94720, USA.

*Email: [roritchie@lbl.gov](mailto:roritchie@lbl.gov) (R.O.R); [luo7128@163.com](mailto:luo7128@163.com) (HY.L)

| **Category** | **Materials** | **Density**  **ρ(g cm^−3^)** | **Modulus**  **E(GPa)** | **Tensile strength**  **σ(MPa)** | **Specific stiffness**  **E/ρ**  **(GPa cm^3^ g^−1^))** | **Specific strength**  **σ/ρ**  **(MPa cm^3^ g^−1^)** | **Ref** |
| --- | --- | --- | --- | --- | --- | --- | --- |
| Natural materials | Moso bamboo | 0.7 | 10 | 150 | 14.29 | 214.29 | [1] |
|  | Pine | 0.5 | 8 | 90 | 16 | 180 | [2] |
|  | Oak | 0.66 | 12 | 70 | 18.18 | 106.06 | [2] |
| Natural fiber composites | Microwave bamboo | 1 | 36.1 | 560 | 36 | 560 | [3] |
|  | Densified bamboo | 1.3 | 59.6 | 770 | 45.8 | 592.3 | [4] |
|  | Densified wood | 1.2 | 35 | 270 | 30 | 225 | [5] |
|  | All-Bio-Based  Laminates | 1.2 | 40 | 250 | 33 | 210 | [6] |
|  | Reconstructed wood | 1.25 | 25 | 200 | 20 | 160 | [7] |
| Natural fiber reinforced materials | SiO2@GO  Bamboo Composites | 1.25 | 22 | 641.6 | 17.6 | 513.3 | [8] |
|  | Bamboo fiber composite | 1.62 | 15 | 517 |  | 319 | [9] |
|  | Cellulose  based materials | / | 26.8 | 350 | / | / | [10] |
|  | Bamboo Steel | 1.35 | 19.6 | 407 | 14.51 | 301.48 | [11] |
|  | Wood/epoxy Composites | 1.3 | 20 | 243 | 15.38 | 186.92 | [12] |
| This work | Densified bamboo | 1.3 | 25 | 278 | 19.23 | 213.85 | / |
|  | TEMPO oxidized densified bamboo | 1.6 | 37 | 661 | 23.13 | 413.13 | / |

**Table S1. Density, modulus, tensile strength and stiffness of natural fiber composites**


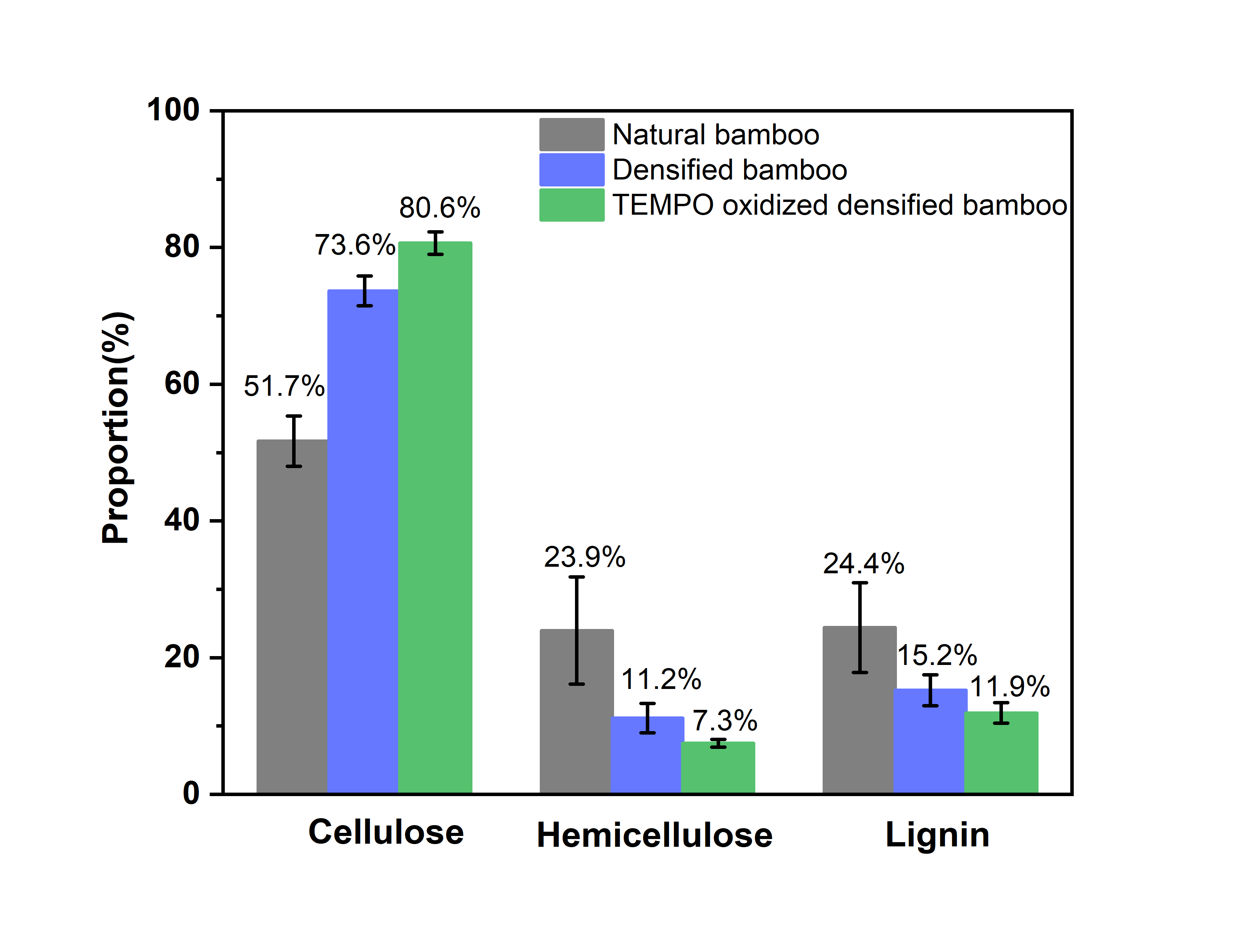


**Fig. S1 Chemical composition of natural bamboo, densified bamboo, and TEMPO-oxidized densified bamboo.** The cellulose, hemicellulose, and lignin contents were determined using the TAPPI T222 standard method. TEMPO oxidation slightly increases the cellulose content while further reducing the hemicellulose and lignin contents, mainly due to the oxidative and mildly alkaline reaction environment that promotes the removal of residual non-cellulosic components without degrading the cellulose framework.


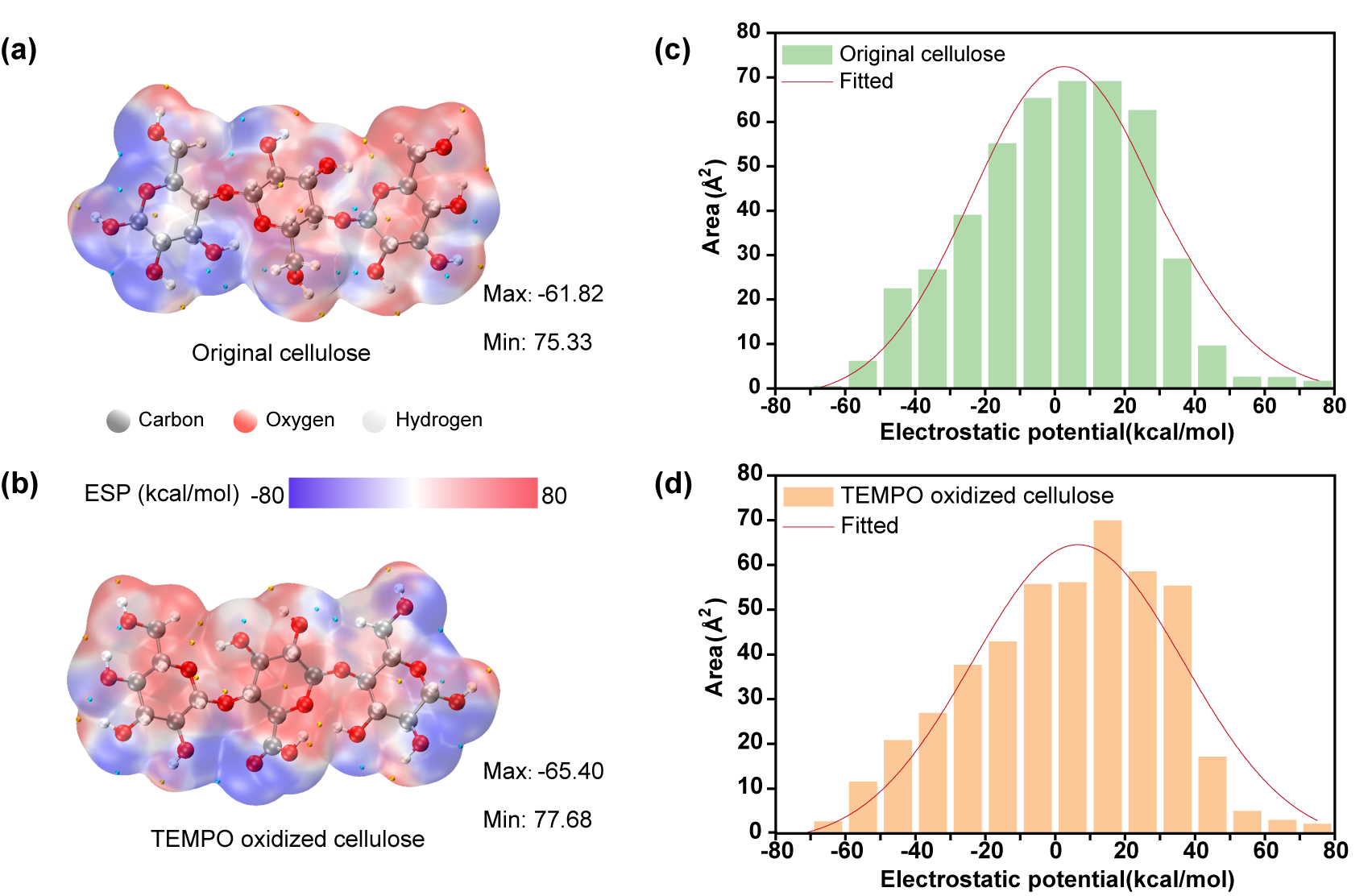


**Fig. S2** **Electrostatic potential (ESP) maps and corresponding surface potential distributions of cellulose before and after TEMPO oxidation.** Surface ESP maps of original cellulose (**a**) and TEMPO-oxidized cellulose (**b**), with false-color gradients ranging from -80 to +80 kcal/mol. Red regions represent electron-rich zones (negative ESP), while blue regions indicate electron-deficient areas (positive ESP). Histogram of surface ESP distribution fitted with Gaussian curves for original cellulose (**c**) and TEMPO-oxidized cellulose (**d**). TEMPO oxidation induces a broader and more positively shifted ESP distribution, indicating enhanced surface polarity and reactivity.


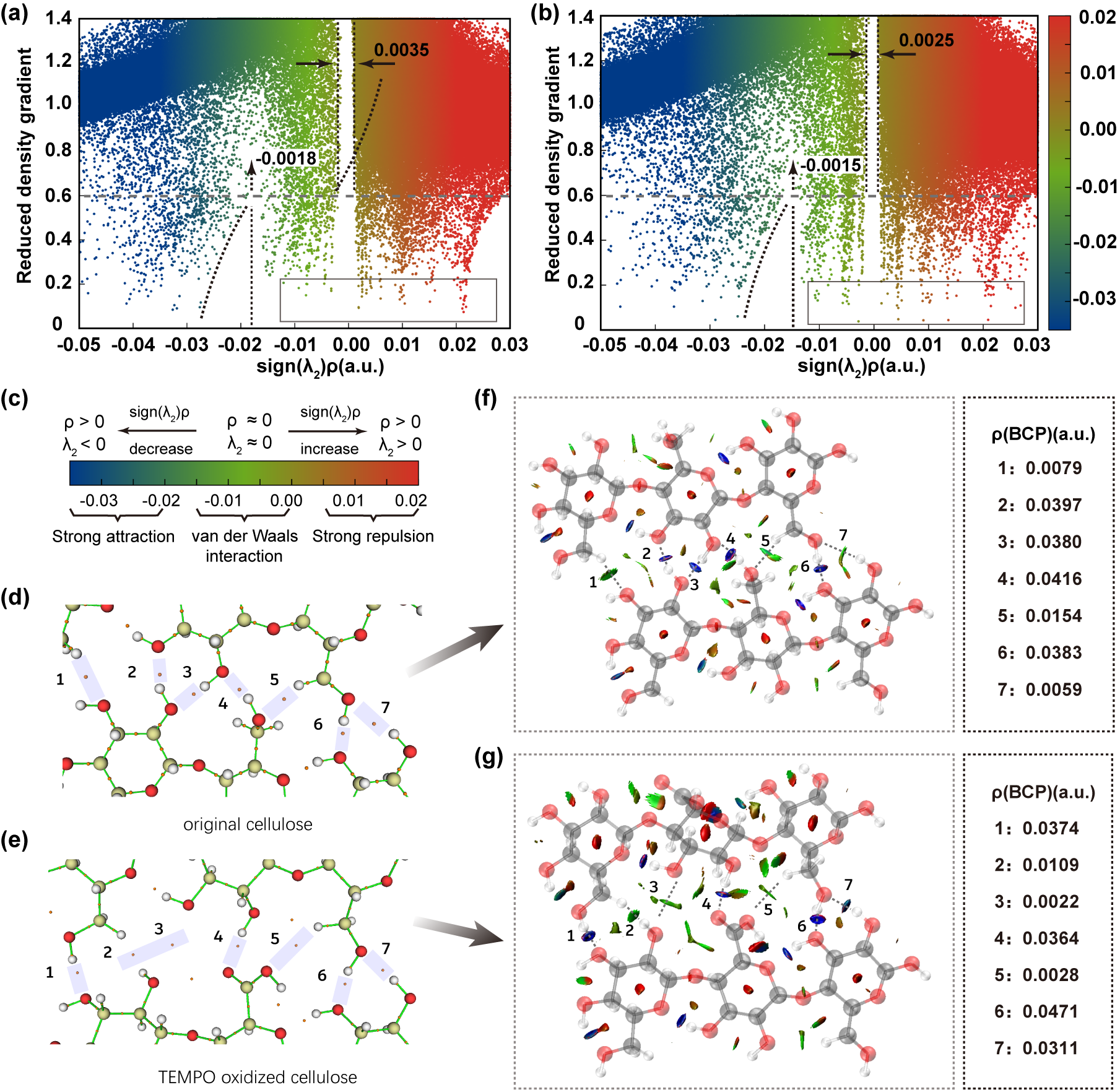


**Fig. S3 Noncovalent interaction (NCI) analysis of cellulose molecules before and after TEMPO oxidation based on reduced density gradient (RDG) and Atoms-in-Molecules (AIM) topology analysis.** RDG scatter plots of original cellulose (**a**) and TEMPO-oxidized cellulose (**b**), where the false-color represents sign(λ_2_)ρ values as defined in (**c**), and the isosurface value is set to 0.6 (gray dashed box). (**d, e**) Critical point analysis of electron density based on AIM theory for original (**d**) and TEMPO-oxidized cellulose (**e**), with labeled bond paths and critical points. Graphical representation of weak interactions in original (**f**) and TEMPO-oxidized cellulose (**g**) based on RDG isosurfaces, where the green surfaces highlight van der Waals interactions and the blue/red areas indicate attractive/repulsive regions. The corresponding electron density values at bond critical points (ρ(BCP), in atomic units) are listed alongside.


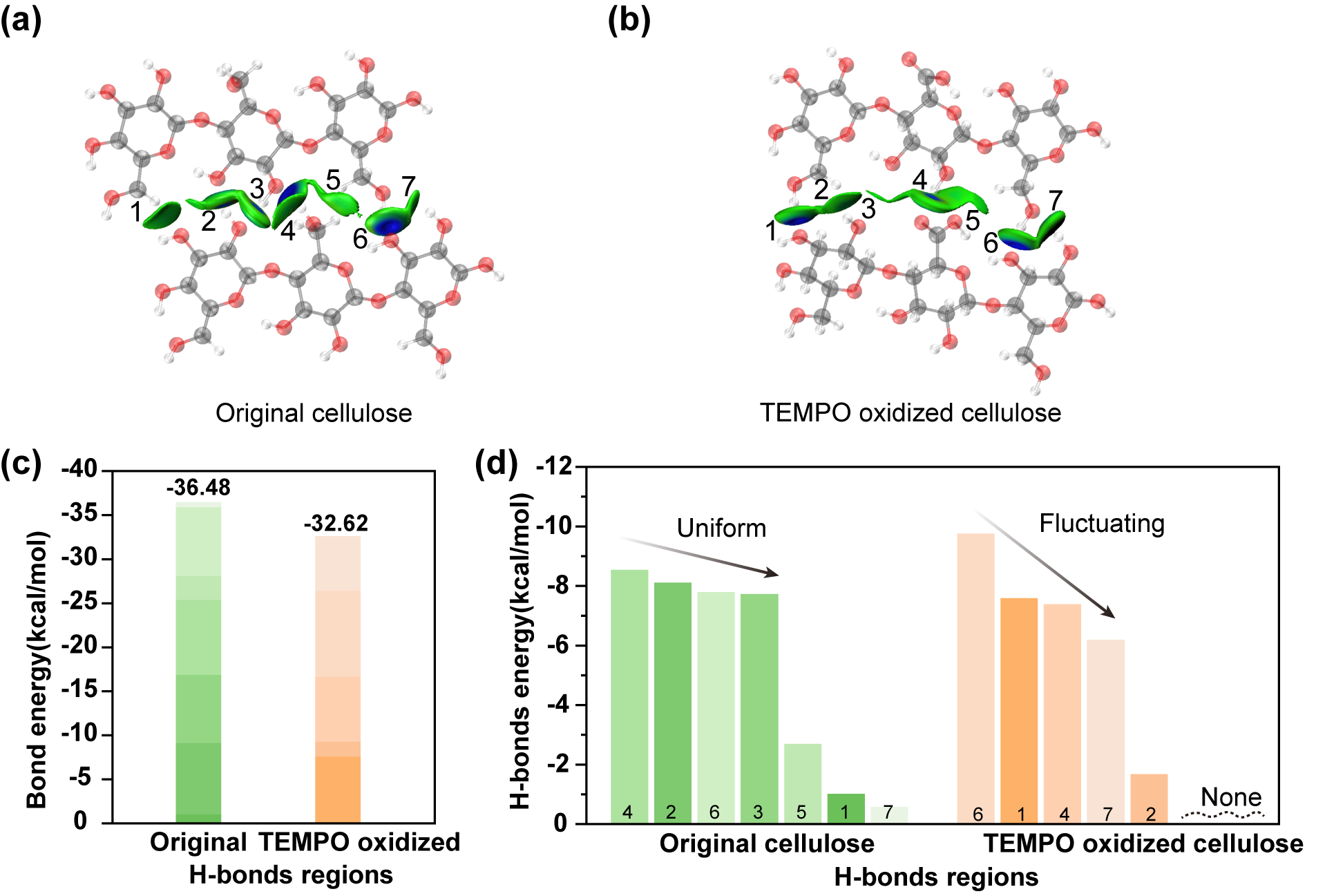


**Fig. S4 Analysis of intermolecular noncovalent interactions and hydrogen bonding in original and TEMPO-oxidized cellulose using the independent gradient model based on Hirshfeld partitioning (IGMH).** IGMH visualizations of noncovalent interactions between cellulose chains in original cellulose (**a**) and TEMPO-oxidized cellulose (**b**), with the isosurface value set at δ_inter_ = 0.002. Green isosurfaces indicate attractive interactions dominated by hydrogen bonding. (**c**) Total hydrogen bond interaction energy in the key bonding regions of the two systems, showing a slight decrease upon TEMPO oxidation. (**d**) Distribution of hydrogen bond energies in each identified interaction region. The hydrogen bonding in original cellulose is relatively uniform and stable, while the TEMPO-oxidized system exhibits more fluctuating and localized hydrogen bond strengths, with some interactions nearly disappearing.

**Table S2. Hydrogen bond energies within original and oxidized cellulose chains.**

| **Interaction area number** | | **1** | **2** | **3** | **4** | **5** | **6** | **7** |
| --- | --- | --- | --- | --- | --- | --- | --- | --- |
| **Cellulose** | **ρ(BCP)(a.u.)** | 0.008 | 0.040 | 0.038 | 0.042 | 0.015 | 0.038 | 0.006 |
|  | **H-bond energy(kcal/mol)** | −1.019 | −8.119 | −7.725 | −8.542 | −2.699 | −7.795 | −0.584 |
| **TEMPO oxidized** | **ρ(BCP)(a.u.)** | 0.037 | 0.011 | 0.002 | 0.036 | 0.003 | 0.047 | 0.031 |
|  | **H-bond energy(kcal/mol)** | −7.595 | −1.679 | / | −7.387 | / | −9.760 | −6.197 |


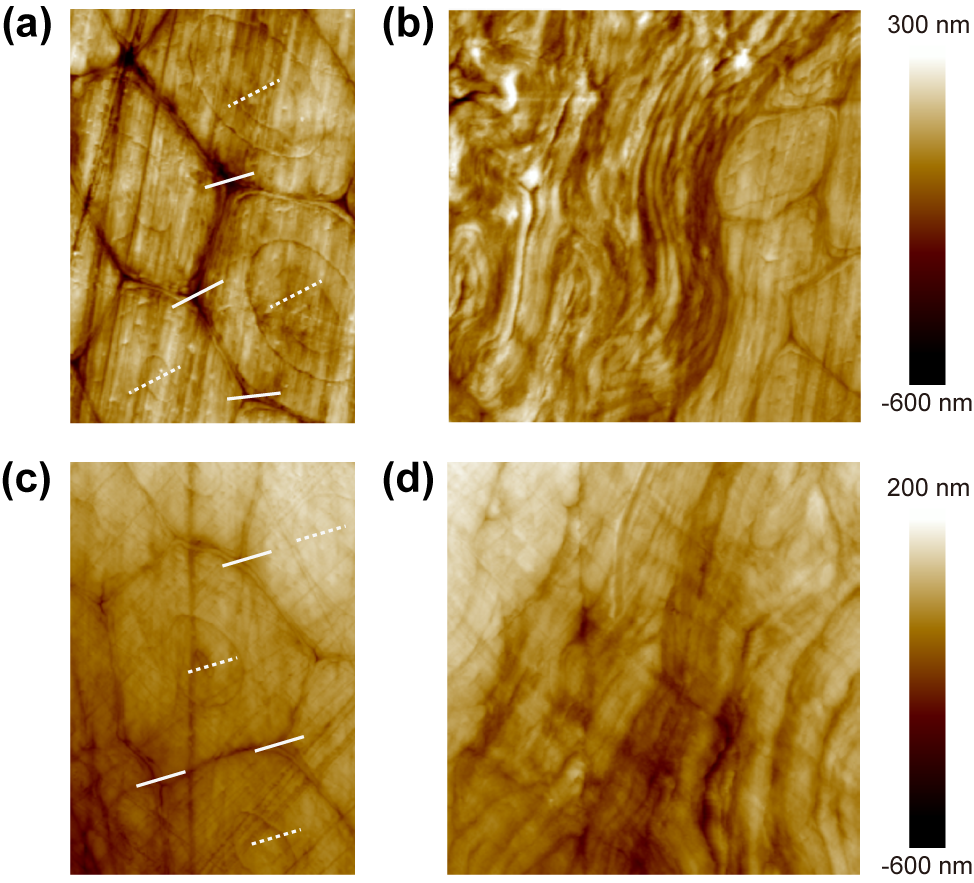


**Fig. S5** **AFM topography images of** **densified bamboo (DB) and TEMPO-oxidized densified bamboo (TODB) showing differences in microstructural morphology.** Surface morphology of densified bamboo with (**a**) corresponding to the microfibril region and (**b**) to the parenchyma cells region. Surface morphology of TEMPO-oxidized densified bamboo with (**c**) corresponding to the microfibril region and (**d**) to the parenchyma cells region. White solid and dashed lines in (**a**) and (**c**) indicate the positions used for extracting modulus profiles shown in Figure 4j and 4l.


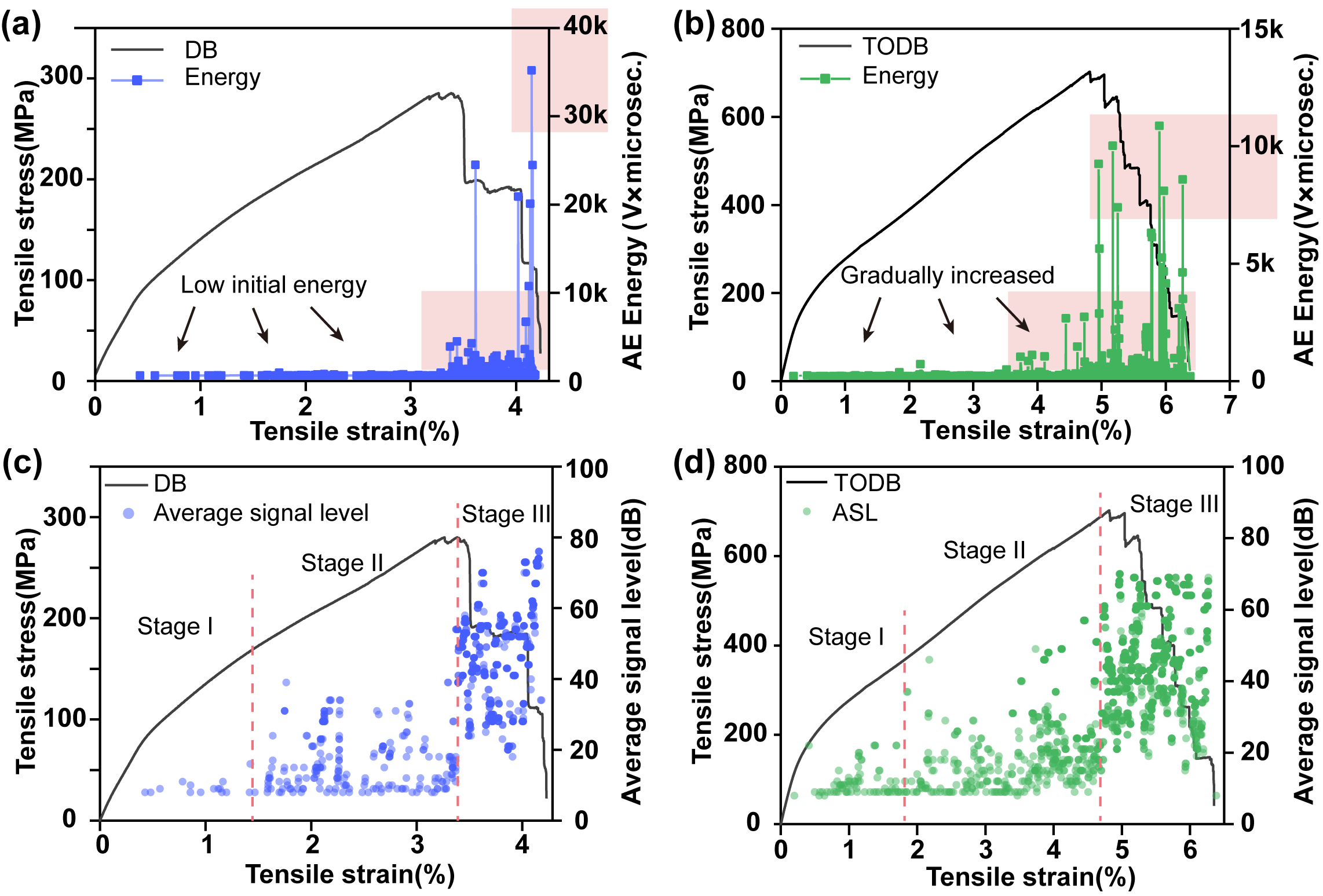


**Fig. S6** **Tensile stress–strain behavior coupled with acoustic emission (AE) analysis for** **densified bamboo (DB) and** **TEMPO-oxidized densified bamboo (TODB).** (a, b) AE energy distribution during tensile testing of (a) densified bamboo and (b) TEMPO-oxidized densified bamboo showing that densified bamboo exhibits low initial AE energy followed by a sudden release near failure, whereas TEMPO-oxidized densified bamboo presents a gradual energy increase across the deformation process. Average signal level (ASL) distribution of AE events for (c) densified bamboo and (d) TEMPO-oxidized densified bamboo revealing three distinct stages of damage evolution during loading. Stage I corresponds to low activity and elastic deformation, Stage II to microdamage accumulation, and Stage III to unstable crack propagation and failure. Red dashed lines mark the boundaries of each stage.


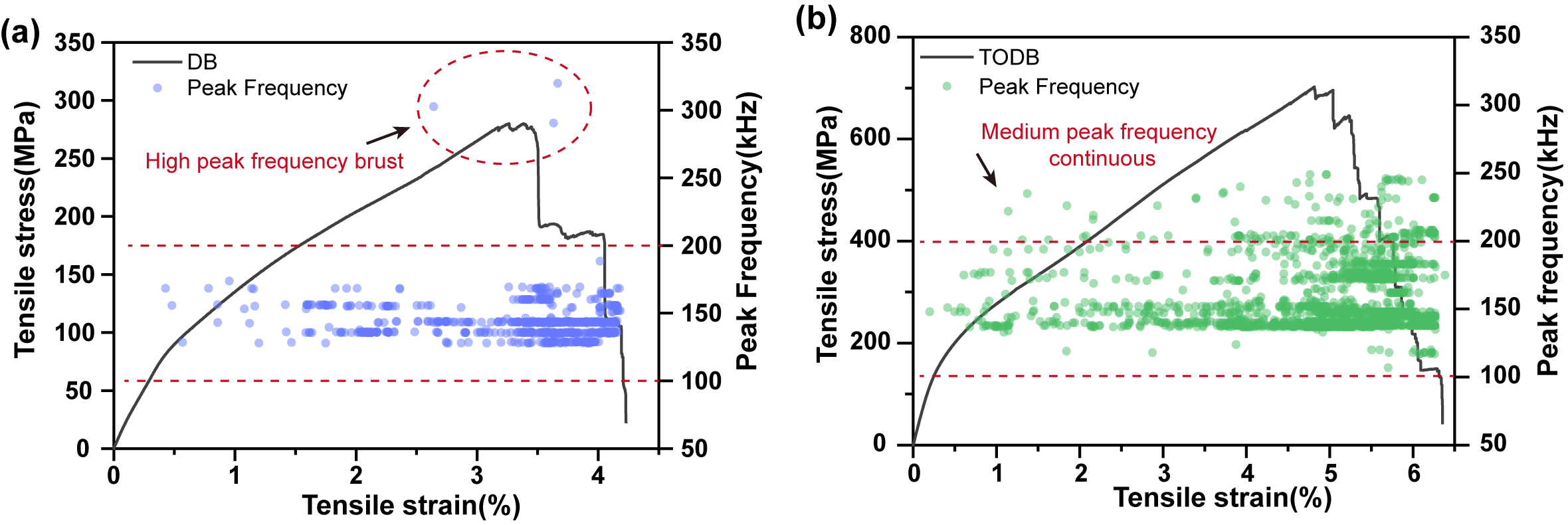


**Fig. S7 Comparison of AE frequency characteristics between (a) densified bamboo and (b) TEMPO oxidized densified bamboo.** For densified bamboo, the AE signals are primarily concentrated within the 100−200 kHz range, corresponding to interfacial debonding events between adjacent fiber bundles. High-frequency bursts around ~300 kHz emerge near final fracture, indicative of abrupt fiber bundle breakage and a brittle failure process. In contrast, TEMPO-oxidized densified bamboo displays continuous AE activity dominated by medium-frequency signals (200−250 kHz) throughout the entire deformation stage. These emissions are associated with progressive microfibril peeling and rupture, signifying a more gradual damage evolution. The absence of sudden high-frequency bursts at failure further confirms the stable, energy-dissipative fracture behavior enabled by TEMPO oxidation.


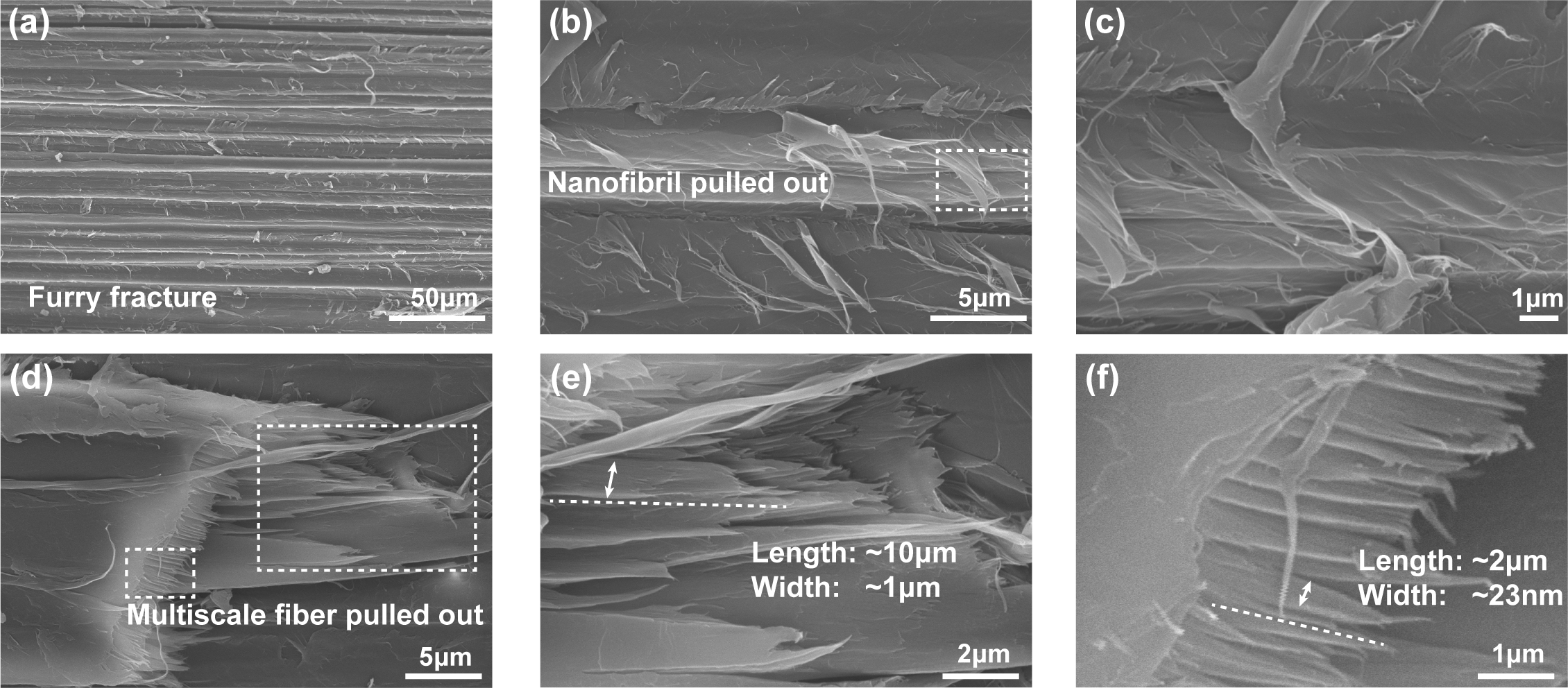


**Fig. S8** **Fracture surface morphology of TEMPO-oxidized densified bamboo (TODB) showing extensive fiber pull-out and interfacial bridging behavior.** (**a**) Low-magnification SEM image showing a characteristic furry fracture surface with aligned microfibrils. (**b, c**) Enlarged views highlighting prominent nanofibril pull-out and bridging structures. (**d**) Overview of multiscale fiber pull-out region. High-magnification images of the boxed areas in (**d**) showing fibers pulled out across different length-scales, from (**e**) microscale fibrils (length ~10 μm, width ~1 μm) to (**f**) nanoscale fibrils (length ~2 μm, width ~23 nm). These observations indicate a hierarchical fracture mechanism in TODB that facilitates energy dissipation through multiscale fiber interactions.


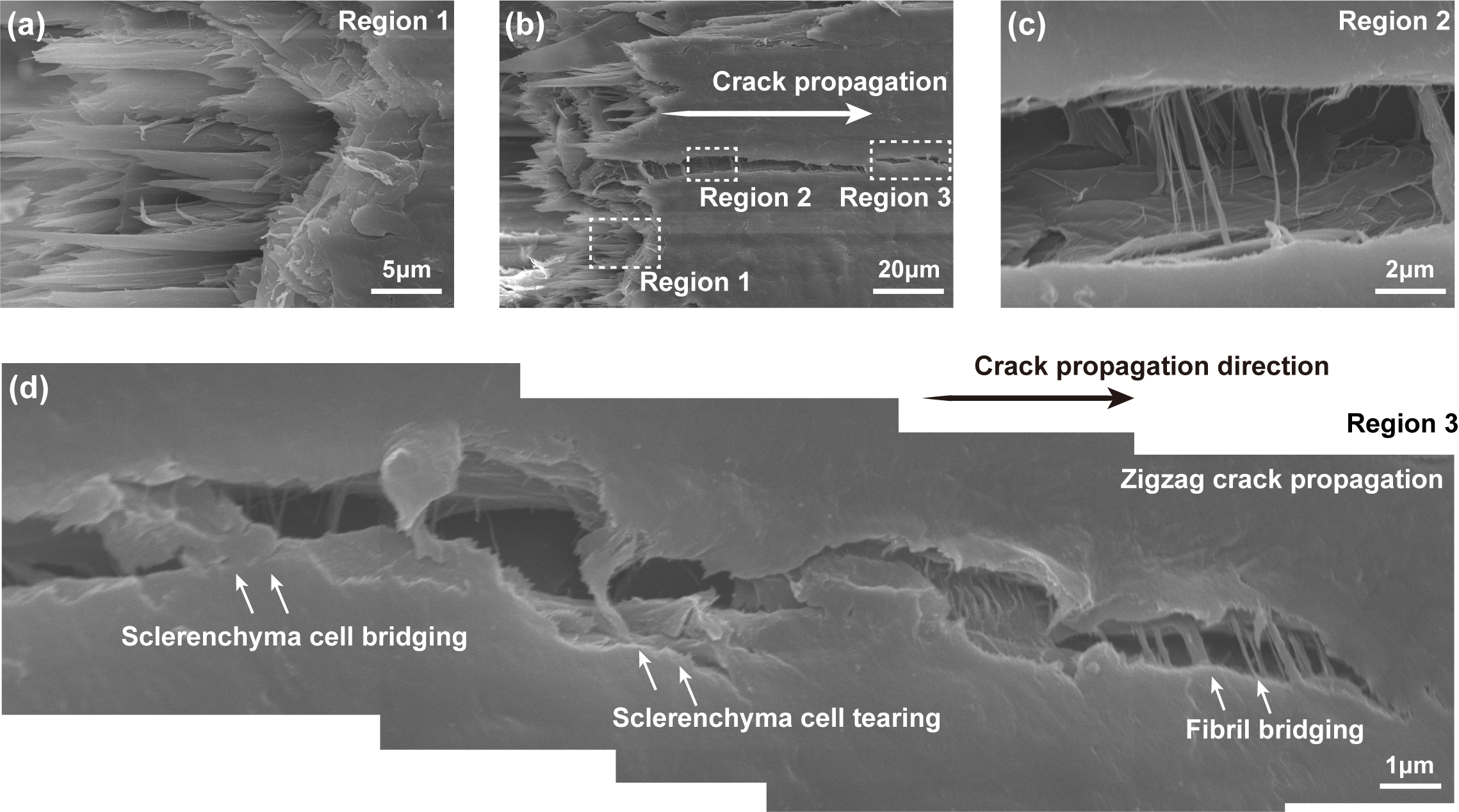


**Fig. S9** **Fracture morphology of TEMPO-oxidized densified bamboo (TODB) revealing crack propagation and toughening mechanisms.** (**a**) High-magnification SEM image of Region 1 showing dense microfibril pull-out and fiber alignment. (**b**) Overview of the crack path highlighting three distinct regions (Region 1, 2, and 3) associated with different crack features. (**c**) Region 2 exhibiting nanoscale fibrillar bridging within interfacial separation. (**d**) Region 3 showing a zigzag crack path with multiple toughening features including sclerenchyma cell bridging, cell tearing, and nanoscale fibril bridging. These multiscale crack deflection and bridging events, which are characteristic of extrinsic toughening, contribute to energy dissipation and fracture resistance in TODB.


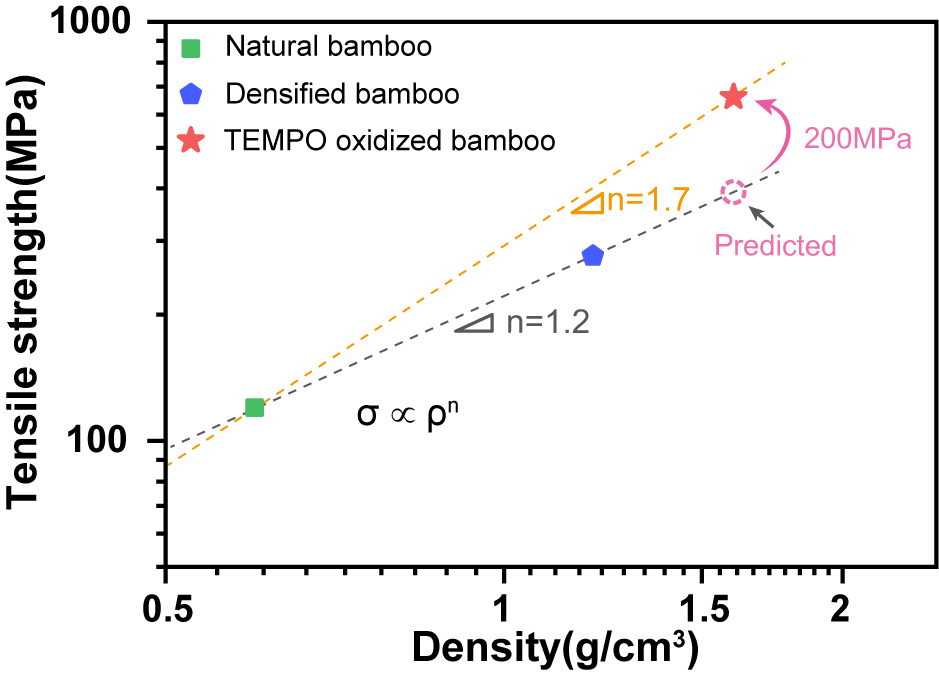


**Fig. S10 Ashby-type strength–density plot comparing natural bamboo (NB), densified bamboo (DB), and TEMPO-oxidized densified bamboo (TODB).** The grey dashed line represents the power-law scaling relationship derived from the natural bamboo to densified bamboo transition. The formula illustrates the power-law relationship (σ ∝ ρ^n^) between tensile strength (σ) and density (ρ), with the exponent *n* representing how strongly tensile strength increases as density increases. For the transition from natural bamboo (NB) to densified bamboo (DB), the tensile strength follows a power-law relationship with density, with an exponent of n≈1.2. This transition involves substantial densification together with pronounced removal of lignin and hemicellulose, and thus provides a conservative baseline that accounts for the combined effects of density increase and conventional chemical composition changes. Specifically, the NB to DB transition reduces lignin and hemicellulose contents by approximately 53% and 38%, respectively, whereas the subsequent DB to TODB step results in a comparatively smaller additional reduction of about 35% and 22%. Despite this only moderate further compositional change induced by TEMPO oxidation, extrapolation from the NB-DB baseline predicts a tensile strength of approximately 400 MPa for TEMPO-oxidized densified bamboo, which is markedly lower than the experimentally measured value of approximately 660 MPa.

**Note S1. Definition of performance metrics in the radar chart**

The radar chart presented in Figure 6i is used to provide a comparative visualization of the comprehensive performance of densified bamboo (DB) and TEMPO-oxidized densified bamboo (TODB). Strength corresponds to tensile strength, and stiffness is defined as the tensile modulus obtained from tensile tests (Figure 5b and c). 1/*D* represents the inverse of bulk density, where *D* is the measured material density. Integrity reflects the structural integrity and damage tolerance of the material and is evaluated based on acoustic emission (AE) statistics, including cumulative AE energy, event density, and Gutenberg–Richter b-value analysis (Figure 5i–j and Figure S6). Sustainability qualitatively reflects the bio-based nature of the material and processing considerations, with particular emphasis on the recyclability and reusability enabled by TEMPO-mediated modification. All parameters were normalized by the maximum value among the compared materials to enable direct comparison and visualization in the radar chart. The radar chart thus provides an integrated assessment of mechanical performance, structural integrity, and sustainability-related attributes of DB and TODB.

**Supplementary References**

1. Nkeuwa, W.N. Zhang, J. Semple, K.E. Chen, M. Xia, Y. and Dai, C., “Bamboo-based composites: A review on fundamentals and processes of bamboo bonding,” *Composites Part B: Engineering* 235 (2022): 109776.

2. Song, J. Chen, C. Zhu, S. et al., “Processing bulk natural wood into a high-performance structural material,” *Nature* 554, no. 7691 (2018): 224–228.

3. Chen, C. Li, Z. Mi, R. et al., “Rapid processing of whole bamboo with exposed, aligned nanofibrils toward a high-performance structural material,” *ACS Nano* 14, no. 5 (2020): 5194–5202.

4. Li, Z. Chen, C. Mi, R. et al., “A strong, tough, and scalable structural material from fast‐growing bamboo,” *Advanced Materials* 32, no. 10 (2020): 1906308.

5. Frey, M. Widner, D. Segmehl, J.S. Casdorff, K. Keplinger, T. and Burgert, I., “Delignified and densified cellulose bulk materials with excellent tensile properties for sustainable engineering,” *ACS Applied Materials & Interfaces* 10, no. 5 (2018): 5030–5037.

6. Frey, M. Schneider, L. Razi, H. et al., “High-performance all-bio-based laminates derived from delignified wood,” *ACS Sustainable Chemistry & Engineering* 9, no. 29 (2021): 9638–9646.

7. Sun, H. Bi, H. Ren, Z. et al., “Hydrostable reconstructed wood with transparency, excellent ultraviolet-blocking performance, and photothermal conversion ability,” *Composites Part B: Engineering* 232 (2022): 109615.

8. Wang, Y.-Y. Li, Y.-Q. Xue, S.-S. et al., “Superstrong, lightweight, and exceptional environmentally stable SiO_2_@GO/bamboo composites,” *ACS Applied Materials & Interfaces* 14, no. 5 (2022): 7311–7320.

9. Lin, Q. Jiang, P. Ren, S. et al., “Advanced functional materials based on bamboo cellulose fibers with different crystal structures,” *Composites Part A: Applied Science and Manufacturing* 154 (2022): 106758.

10. Hu, J. Zhang, Y. He, Y. et al., “Transformation of bamboo: From multiscale fibers to robust and degradable cellulose‐based materials for plastic substitution,” *Small* (2025): 2411339.

11. Wang, Y.-Y. Wang, X.-Q. Li, Y.-Q. et al., “High-performance bamboo steel derived from natural bamboo,” *ACS Applied Materials & Interfaces* 13, no. 1 (2021): 1431–1440.

12. Huang, Y. Jiang, K. He, Y. et al., “A natural lignification inspired super‐hard wood‐based composites with extreme resilience,” *Advanced Materials* (2025): 2502266.
